# Molecular classification of a complex structural rearrangement of the *RB1* locus in an infant with sporadic, isolated, intracranial, sellar region retinoblastoma

**DOI:** 10.1186/s40478-021-01164-z

**Published:** 2021-04-07

**Authors:** Kathleen M. Schieffer, Alexander Z. Feldman, Esko A. Kautto, Sean McGrath, Anthony R. Miller, Maria Elena Hernandez-Gonzalez, Stephanie LaHaye, Katherine E. Miller, Daniel C. Koboldt, Patrick Brennan, Benjamin Kelly, Amy Wetzel, Vibhuti Agarwal, Margaret Shatara, Suzanne Conley, Diana P. Rodriguez, Rolla Abu-Arja, Ala Shaikhkhalil, Matija Snuderl, Brent A. Orr, Jonathan L. Finlay, Diana S. Osorio, Annie I. Drapeau, Jeffrey R. Leonard, Christopher R. Pierson, Peter White, Vincent Magrini, Elaine R. Mardis, Richard K. Wilson, Catherine E. Cottrell, Daniel R. Boué

**Affiliations:** 1 The Steve and Cindy Rasmussen Institute for Genomic Medicine, Abigail Wexner Research Institute At Nationwide Children’s Hospital, 575 Children’s Crossroad, Columbus, OH 43215 USA; 2 Department of Pathology, Northwestern University Feinberg School of Medicine, Chicago, IL USA; 3 Department of Pediatrics, The Ohio State University College of Medicine, Columbus, OH USA; 4 Division of Hematology/Oncology, Department of Pediatrics, Nemours Children’s Health System, Orlando, FL USA; 5 Division of Hematology/Oncology, Department of Pediatrics, Washington University School of Medicine in St. Louis, St. Louis, MO USA; 6 Division of Hematology, Oncology, and Bone Marrow Transplant, Nationwide Children’s Hospital, Columbus, OH USA; 7 Department of Radiology, Nationwide Children’s Hospital, Columbus, OH USA; 8 Division of Gastroenterology & Hepatology & Nutrition, Nationwide Children’s Hospital, Columbus, OH USA; 9 Department of Pathology, New York University Langone Health, New York City, NY USA; 10 Department of Pathology, St. Jude Children’s Research Hospital, Memphis, TN USA; 11 Division of Hematology and Oncology, The Ohio State University College of Medicine, Columbus, OH USA; 12 Departments of Pediatrics and Radiation Oncology, The Ohio State University College of Medicine, Columbus, OH USA; 13 Division of Neurosurgery, Nationwide Children’s Hospital, Columbus, OH USA; 14 Department of Neurosurgery, The Ohio State University College of Medicine, Columbus, OH USA; 15 Department of Pathology and Laboratory Medicine, Nationwide Children’s Hospital, Columbus, OH USA; 16 Department of Pathology, The Ohio State University College of Medicine, Columbus, OH USA; 17 Department of Biomedical Education & Anatomy, The Ohio State University, Columbus, OH USA

**Keywords:** Intracranial retinoblastoma, Sellar-suprasellar retinoblastoma, RB1, Structural variation, DNA array-based methylation, SMRT sequencing, PacBio

## Abstract

Retinoblastoma is a childhood cancer of the retina involving germline or somatic alterations of the RB Transcriptional Corepressor 1 gene, *RB1*. Rare cases of sellar-suprasellar region retinoblastoma without evidence of ocular or pineal tumors have been described. A nine-month-old male presented with a sellar-suprasellar region mass. Histopathology showed an embryonal tumor with focal Flexner-Wintersteiner-like rosettes and loss of retinoblastoma protein (RB1) expression by immunohistochemistry. DNA array-based methylation profiling confidently classified the tumor as pineoblastoma group A/intracranial retinoblastoma. The patient was subsequently enrolled on an institutional translational cancer research protocol and underwent comprehensive molecular profiling, including paired tumor/normal exome and genome sequencing and RNA-sequencing of the tumor. Additionally, Pacific Biosciences (PacBio) Single Molecule Real Time (SMRT) sequencing was performed from comparator normal and disease-involved tissue to resolve complex structural variations. RNA-sequencing revealed multiple fusions clustered within 13q14.1-q21.3, including a novel in-frame fusion of *RB1-SIAH3* predicted to prematurely truncate the RB1 protein. SMRT sequencing revealed a complex structural rearrangement spanning 13q14.11-q31.3, including two somatic structural variants within intron 17 of *RB1*. These events corresponded to the *RB1-SIAH3* fusion and a novel *RB1* rearrangement expected to correlate with the complete absence of RB1 protein expression. Comprehensive molecular analysis, including DNA array-based methylation profiling and sequencing-based methodologies, were critical for classification and understanding the complex mechanism of *RB1* inactivation in this diagnostically challenging tumor.

**Supplementary Information:**

The online version contains supplementary material available at 10.1186/s40478-021-01164-z.

## Introduction

Retinoblastoma is a childhood cancer predominately occurring intraocularly, with an estimated prevalence of 1 per 16,000–18,000 live births and approximately 8,000 new cases per year [3]. It is characterized by biallelic inactivation of the RB Transcriptional Corepressor 1 gene (*RB1*) which encodes the retinoblastoma protein (RB1), a well-established tumor suppressor that interacts with the E2F family of transcription factors to negatively regulate the cell cycle. As described by Knudson’s “two-hit hypothesis,” individuals with hereditary retinoblastoma present with a germline heterozygous alteration in *RB1*. Somatic inactivation of the second *RB1* allele results in the development of retinoblastoma [14]. Approximately 40% of individuals with retinoblastoma have a hereditary form, commonly associated with bilateral retinoblastoma (i.e., dual primary tumors presenting in both eyes) early in life [21, 22]. Unilateral retinoblastoma most frequently arises in individuals with biallelic somatic alterations in the developing retina [3]. Rarely, individuals may develop bilateral retinoblastoma together with synchronous or asynchronous intracranial pineal and/or sellar region tumors, i.e. involving the sella turcica/pituitary gland area (so-called trilateral and/or “quadrilateral” retinoblastoma) [6]. Intracranial central nervous system (CNS) tumors are identified prior to ocular retinoblastoma diagnosis in only 3% of cases [5, 35].

Intracranial sellar-suprasellar region retinoblastoma without evidence of ocular or pineal tumor is an exceedingly rare occurrence, with only a single report described in the literature to our knowledge [13]. An early case series reported an infant with isolated suprasellar tumor, without retinal involvement [13]. This patient was noted to have a positive family history, including bilateral retinoblastoma in a sibling and paternal unilateral retinoblastoma. A second report described an infant with biallelic somatic *RB1* inactivating alterations (LRG_517t1:c.T494A;p.Leu165*, LRG_517t1:c.717dup;p.Lys249*) in the absence of a germline *RB1* alteration, who presented with an ectopic, intracranial, sellar-suprasellar region retinoblastoma, but also had a smaller similar-appearing pineal mass detected on imaging [23]. In the latter case, the diagnosis was supported by DNA array-based methylation profiling on the sellar region tumor; the pineal region tumor was not sampled. Additional data are necessary to better understand the frequency and genomic mechanisms associated with isolated, ectopic, intracranial, sellar-suprasellar region retinoblastoma.

The genomic landscape of retinoblastoma is diverse and may result from a spectrum of events, including single nucleotide variations (SNVs), small insertion-deletion (indel) events, splice site alterations, copy number alterations (CNAs), loss of heterozygosity (LOH), and promoter hypermethylation [15, 21, 22]. Highly penetrant germline alterations consist of predominately loss-of-function variants (e.g. nonsense, frameshift, canonical splice site) predicted to encode a premature stop of translation in the protein [21]. Low penetrance alterations are thought to reduce gene expression or partially inactivate the RB1 protein and may be associated with promoter variants, non-canonical splice site alterations, missense variants, and small non-frameshift deletions [8, 32]. In an estimated 60–70% of hereditary and non-heritable tumors, the initial *RB1* alteration is followed by LOH at the 13q14.2 locus, encompassing *RB1* [21]. Chromothripsis involving 13q14.2 has been described as an exceedingly rare mechanism of *RB1* inactivation in individuals with somatic disease [15, 20]. In three retinoblastoma patients, chromothripsis disrupted the *RB1* locus resulting in a gene fusion, and immunohistochemical (IHC) staining demonstrated complete absence of RB1 protein expression in the tumor [20]. Additionally, genome sequencing of retinoblastoma tumors without a previously identified *RB1* alteration revealed large structural rearrangements encompassing the *RB1* locus with variable complexity [2]. Beyond the *RB1* gene, amplification of the proto-oncogene *MYCN* has been reported in a small subset (~ 2%) of sporadic retinoblastoma cases without *RB1* alterations [24].

Herein, we describe a patient with a sporadic, isolated, intracranial, sellar-suprasellar region retinoblastoma with diagnostic refinement by DNA array-based methylation profiling as well as comprehensive genomic profiling of germline and somatic tissues. Complex structural rearrangement of 13q14.11-q31.3 was identified, including two somatic *RB1* gene rearrangements. Considerations for therapeutic management are presented.

## Case Presentation

### Clinical history and diagnostic work-up

**Fig. 1:**
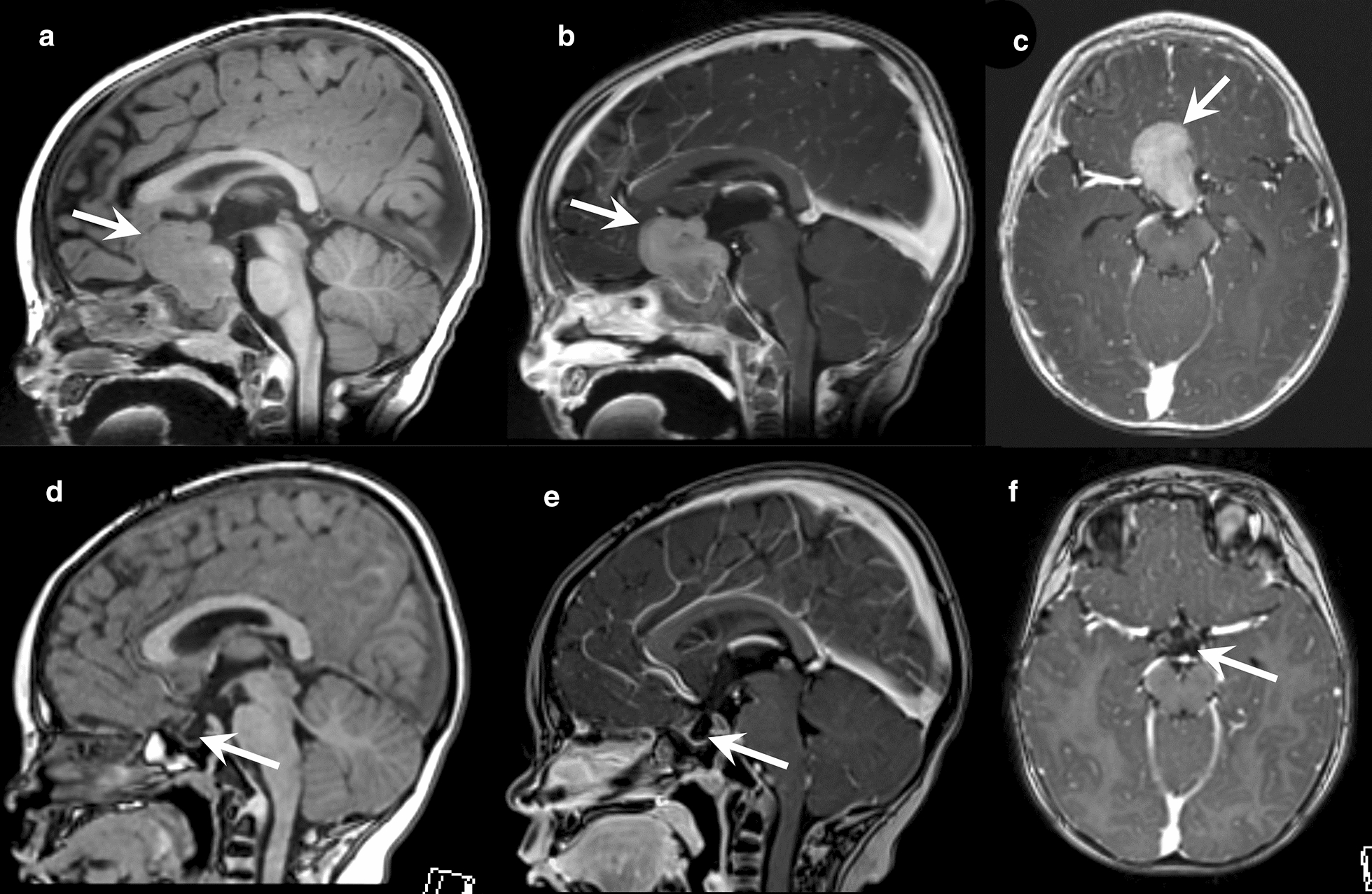
*At diagnosis*: Pre contrast sagittal T1-W image **(a)**, post contrast sagittal T1-W image **(b)**, and post contrast axial T1-W image **(c)** show a large lobulated avidly enhancing mass that fills and expands the sella, and extends to the suprasellar cistern (arrows). This mass is separated from the optic nerves and optic chiasm, and contacts the circle of Willis vessels, which preserve normal caliber. *At completion of therapy*: Pre contrast sagittal T1-W image **(d)**, post contrast sagittal T1-W image **(e)**, and post contrast axial T1-W image **(f)** show complete resolution of the sellar, suprasellar mass with no evidence of residual tumor, an empty sella (arrows), and normal surrounding structures

A nine-month-old male with no significant past medical history initially presented with two months of worsening fussiness and two days of vomiting. His physical exam was unremarkable and there was no history of polyuria or polydipsia. A magnetic resonance imaging (MRI) of the brain revealed a 3.2 × 3.0 × 2.6 cm lobular sella-suprasellar mass, with avid contrast enhancement (Fig. 1a-c). The mass was separate from the optic chiasm, eroded the dorsum sella, and extended into the subfrontal region. Intralesional hemorrhage was noted as well (not shown). An MRI of the spine was negative for metastases. The primary radiologic differential was germ cell tumor vs. atypical teratoid/rhabdoid tumor. An endocrinology workup confirmed central adrenal insufficiency requiring maintenance and stress doses of hydrocortisone. A craniotomy was performed, and the tumor was found to be adherent to surrounding critical structures. A subtotal resection was achieved.

**Fig. 2:**
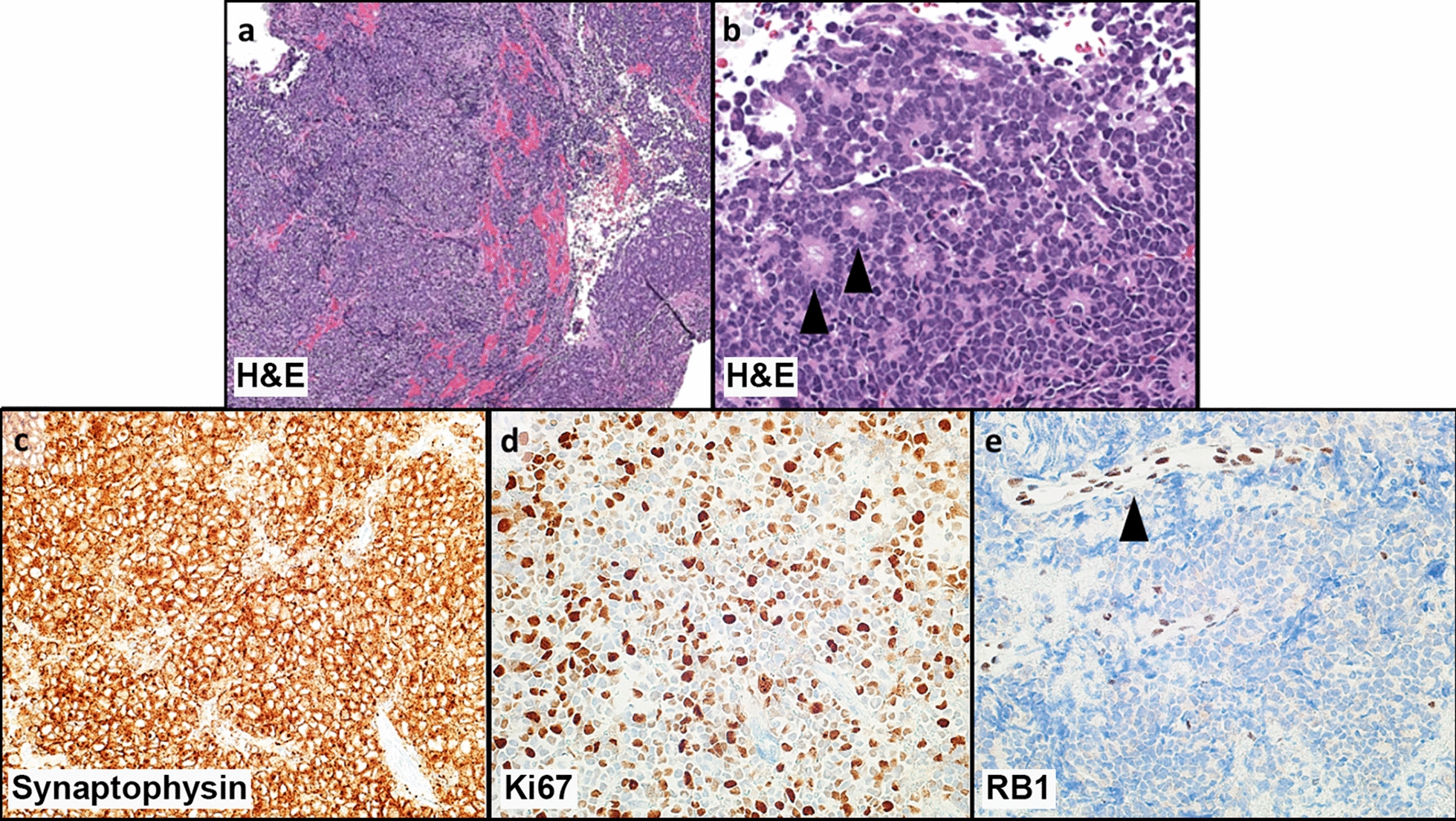
Tumor histology and immunohistochemistry. **a** Hematoxylin and eosin (H&E) sections reveal an embryonal neoplasm composed of mostly patternless sheets of small tumor cells, 40×. **b** At higher magnification, the tumor cells are poorly differentiated, with nuclear pleomorphism, some nuclear molding, and occasional apoptotic bodies and mitoses. Rare Flexner-Wintersteiner-like rosettes were seen focally (arrowheads) 200×. **c** Synaptophysin immunohistochemistry (IHC) is diffusely, strongly positive, 200×. **d** Ki-67 tumor nuclear labeling index is approximately 40–50%, 200×. **(e)** IHC for RB Transcriptional Corepressor 1 (RB1) protein demonstrates loss of constitutive nuclear staining in tumor cell nuclei, but retained normal staining in vascular endothelial cell nuclei (arrowhead), 200×

Hematoxylin and eosin (H&E) stained sections demonstrated a small cell embryonal neoplasm characterized by sheets of poorly differentiated cells with nuclear pleomorphism, nuclear molding, and occasional apoptotic bodies and mitoses (Fig. 2a). Rare rosettes were appreciated, composed of a single layer of tumor cells with short cytoplasmic processes extending into an otherwise empty lumen (Flexner-Wintersteiner-like rosettes, Fig. 2b). Rhabdoid inclusions, abundant neuropil, perivascular rosettes, microvascular proliferation, necrosis, primitive neural tubes, and/or glandular structures were not seen.

Synaptophysin IHC demonstrated diffuse and strong positivity (Fig. 2c). The Ki-67 labeling index was approximately 40–50% (Fig. 2d). By IHC, tumor cells showed only focal, weak staining or were negative for Olig2, BCL-6 interacting corepressor protein (BCOR), nuclear protein in testis family member 1 protein (NUTM1), neuronal nuclei protein (Neu-N), neurofilament, glial fibrillary acid protein (GFAP), p53, SALL4, epithelial membrane antigen (EMA), and lin-28 homolog A (Lin28A). Tumor cells retained both integrase interactor 1 (INI-1) and transcription activator BRG1 (BRG1). Fluorescence *in-situ* hybridization (FISH) was negative for rearrangements of *BCOR*, capicua transcriptional repressor gene (*CIC*)*,* or Ewing sarcoma breakpoint region 1 (*EWSR1*) and negative for amplification of the C19MC locus at 19q13.42. DNA array-based methylation profiling confidently classified the tumor as pineoblastoma group A/intracranial retinoblastoma (calibrated score: 0.99) [1]. Subsequently, IHC staining for RB1 protein was performed, and showed complete loss of normal constitutive nuclear expression in tumor cells, with retained expression in tumor vascular endothelial nuclei (Fig. 2e).

The patient was enrolled on the Head Start 4 clinical trial (NCT02875314) with the diagnosis of CNS embryonal tumor, not otherwise specified (NOS) and underwent five induction cycles of intensive chemotherapy with 12 g/m^2^ (400 mg/kg) of methotrexate. Radiologic evaluation at the completion of therapy demonstrated no evidence of residual tumor (Fig. 1d-f). He was randomized into tandem autologous hematopoietic stem-cell rescues with carboplatin and thiotepa. His post-operative and overall clinical course were complicated by partial pituitary dysfunction (hypothyroidism, adrenal insufficiency), bacterial and fungal sepsis, pneumatosis coli and feeding challenges, and compression fracture. However, the patient remained without significant CNS deficits.

He underwent periodic sedated ophthalmologic evaluations to visualize the retinae during scheduled surveillance MRIs, which showed no evidence of retinal disease. Retinal surveillance was discontinued at 2 years of age as development of classic retinoblastoma would be unlikely after this age. He is currently 3 years old and 1.5 years off-therapy and doing well with continued follow-up by gastroenterology for his feeding challenges. He tolerates full enteral calories via G-tube, has started making steady progress in improving his oral intake, and is gaining weight well.

## Materials and methods

The patient was enrolled as part of an Institutional Review Board (IRB) approved study at The Steve and Cindy Rasmussen Institute for Genomic Medicine (IGM) at Nationwide Children’s Hospital (NCH). Informed consent was provided by the patient’s parents for comprehensive genomic analysis. Peripheral blood mononuclear cells (PBMCs) were collected by routine venipuncture for genomic DNA extraction. Snap-frozen tumor tissue was obtained for DNA and RNA extraction (estimated 80% tumor cellularity). Full materials and methods can be found in the Supplementary Information (Additional File 1: Materials and Methods).

### Molecular characterization of intracranial retinoblastoma reveals RB1 loss-of-function due to gene fusion

**Fig. 3:**
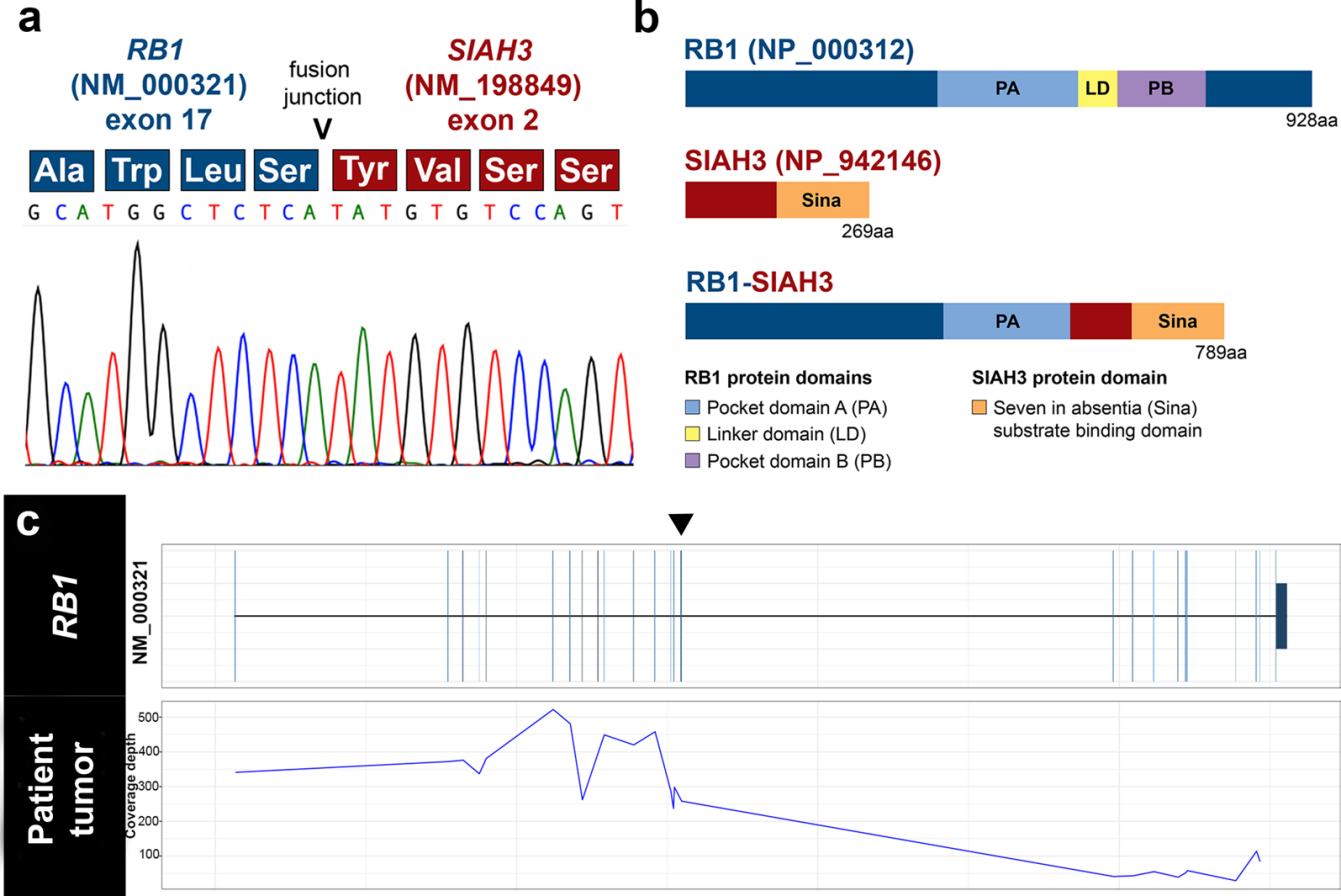
Molecular characterization of an in-frame *RB1-SIAH3* fusion in a pediatric intracranial retinoblastoma. **a** Sanger sequencing chromatogram of the in-frame *RB1-SIAH3* gene fusion. The corresponding amino acid sequence is described above the chromatogram. **b** Protein domains corresponding to the *RB1-SIAH3* fusion, demonstrating loss of the RB1 linker domain and pocket domain B. **c** RNA sequencing read coverage for *RB1* (NM_000321) binned per exon*.* A dramatic loss of *RB1* expression occurs between exons 17 (black arrowhead) and 18 at the fusion breakpoint

Comprehensive molecular profiling, including paired tumor/normal genome sequencing (GS) and enhanced exome sequencing (eES) were performed using Illumina next-generation sequencing (NGS) to evaluate germline variations, somatic SNVs, small indels, structural variations (SVs), and CNAs. Notably, we did not identify any pathogenic germline alterations in well-established cancer predisposition genes [36], with the *RB1* and *DICER1* loci assessed at moderate depth within the GS germline data (> 70 × and > 100 × coverage, respectively) and at high depth in eES germline data (> 400 × and ~ 450 × coverage, respectively). Although the tumor did not harbor any clearly medically meaningful somatic SNVs or small indels, we identified a gain on chromosomal arm 1q and a focal deletion on 13q distal to, but not encompassing, *RB1* (Additional File 1: Fig. S1a-b). This focal deletion (hg19: chr13:57,297,261–58,439,492) does not contain any genes associated with cancer. We did not identify any CNAs or LOH associated with the *RB1* locus. RNA-sequencing of the tumor tissue revealed two gene fusion events, clustered within chromosomal bands 13q14.1-q21.3, *RB1-SIAH3* and *ZC3H13-KLHL1*, and two interchromosomal fusions between chromosomes 10 and 13 (*DLEU1-DNAJC12* and *RBP3-TPT1*) (Table 1). *ZC3H13-KLHL1, DLEU1-DNAJ12,* and *RBP3-TPT1* are not recurrent fusions in cancer nor are the genes involved in these events recurrently altered in pediatric cancers. Therefore, the association of these events with tumorigenesis is unknown. We performed reverse transcriptase polymerase chain reaction (RT-PCR) followed by Sanger sequencing to confirm the *RB1-SIAH3* fusion and verify the inferred translational protein frame. The in-frame fusion event involved exon 17 of *RB1* (NM_000321) and exon 2 of *SIAH3* (NM_198849), resulting in a 789 amino acid chimeric protein which is predicted to prematurely truncate *RB1* approximately 60% of the way through the translated protein (Fig. 3a). The fusion retains pocket domain A of the RB1 protein but demonstrates loss of both the linker domain and pocket domain B (Fig. 3b). Interaction of pocket domains A and B with the linker domain is important for supporting the tumor suppressor role of RB1 [9]. Read coverage of this *RB1* transcript (NM_000321), as derived from RNA sequencing, revealed minimal reads supporting full-length *RB1*, with a loss of sequence read depth at the fusion breakpoint (exon 17) through the 3′ terminus (Fig. 3c). The loss of full-length *RB1* expression is consistent with the absence of RB1 protein demonstrated in the tumor by IHC (Fig. 2e). Given an estimated tumor content of 80% in the sequenced tumor tissue, the low number of detected full-length transcript reads likely correspond to admixed normal brain and connective tissue, as demonstrated by retained RB1 expression in tumor vascular endothelial nuclei by IHC (Fig. 2e).

**Table 1:** Gene fusions surrounding the *RB1* locus at 13q14.1-q21.3

Fusion	5′ gene partner	5′ gene partner coordinates (GRCh38), exon	Cytoband	3′ gene partner	3′ gene partner coordinates (GRCh38), exon	Cytoband
*RB1-SIAH3*	*RB1* (NM_000321)	chr13:48,381,443, exon 17	13q14.2	*SIAH3* (NM_198849)	chr13:45,784,057, exon 2	13q14.13
*ZC3H13-KLHL1*	*ZC3H13* (NM_001076788)	chr13:46,003,139, exon 8	13q14.13	*KLHL1* (NM_020866)	chr13:69,975,802, exon 2	13q21.33
*DLEU1-DNAJC12*	*DLEU1* (NR_109974)	chr13:50,433,550, exon 4	13q14.3	*DNAJC12* (NM_021800)	chr10:67,823,392, exon 2	10q21.3
*RBP3-TPT1*	*RBP3* (NM_002900)	chr10:47,349,271, exon 1	10q11.22	*TPT1* (NM_003295)	chr13:45,341,148, exon 1	13q14.13

*RB1,* RB Transcriptional Corepressor 1; *SIAH3,* Siah E3 Ubiquitin Protein Ligase Family Member 3; *ZC3H13,* Zinc Finger CCCH-Type Containing 13; *KLHL1,* Kelch-like 1 gene; *DLEU1,* Deleted In Lymphocytic Leukemia 1; *DNAJC12,* DnaJ Heat Shock Protein Family (Hsp40) Member C12; *RBP3,* Retinol Binding Protein 3; *TPT1*, Tumor Protein, Translationally-Controlled 1

### Complex chromosome 13q structural rearrangement identified through SMRT sequencing methodologies

**Fig. 4:**
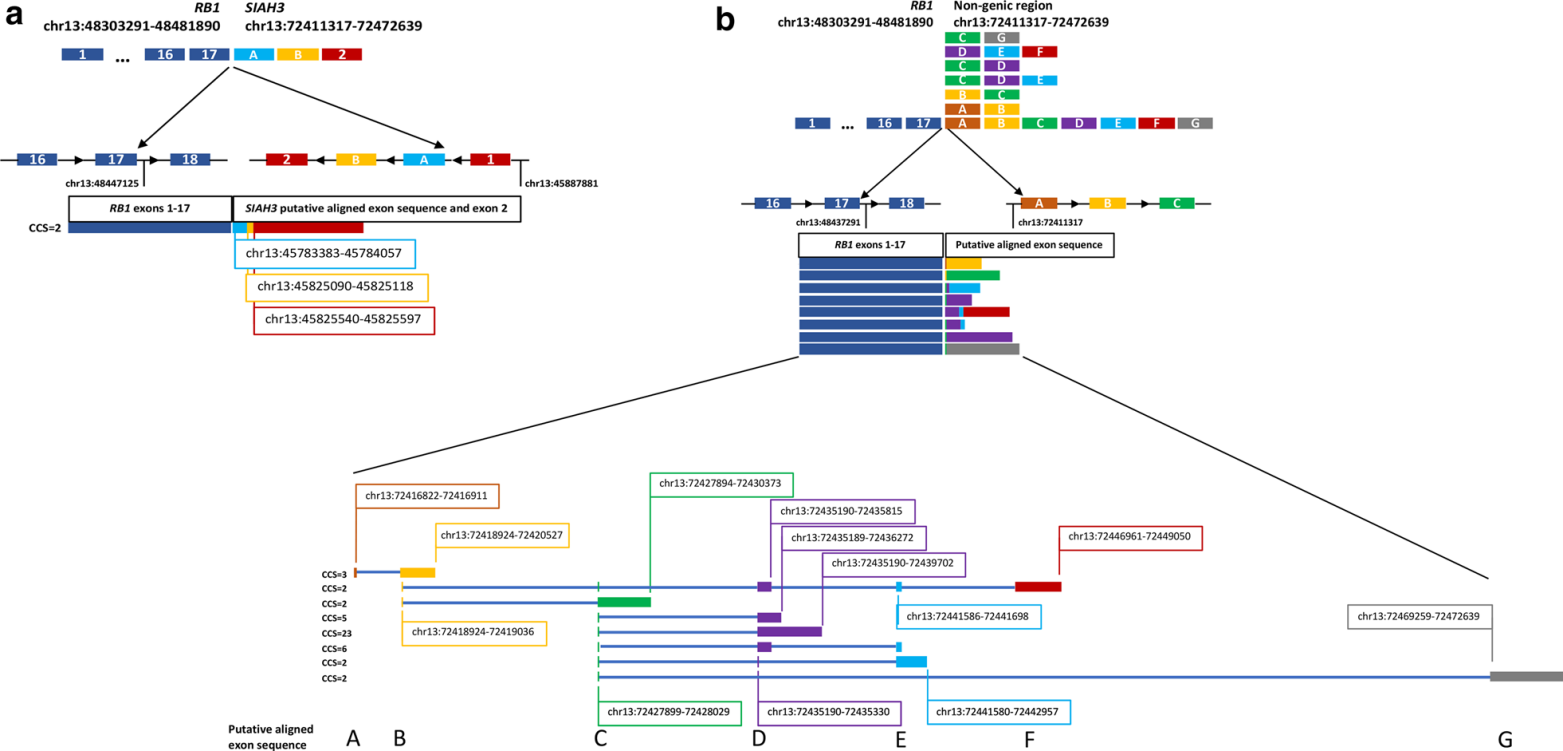
PacBio SMRT sequencing by Iso-Seq resolves *RB1* transcript isoform structure and diversity. **a** SMRT sequencing of tumor-derived RNA aligned to GRCh38 identified a novel *RB1-SIAH3* fusion which included putative aligned exon sequence derived from intron 1 of *SIAH3* prior to the fusion junction. **b** Multiple transcript isoforms of an *RB1-*intergenic fusion. The intergenic sequence aligned to a non-genic region of the genome and resulted in the formation of putative aligned exon sequence supported by flanking splice donor and acceptor sequence. Each putative aligned exon is designated as A-G. The supporting number of circular consensus sequencing (CCS) reads are shown for each isoform

**Fig. 5:**
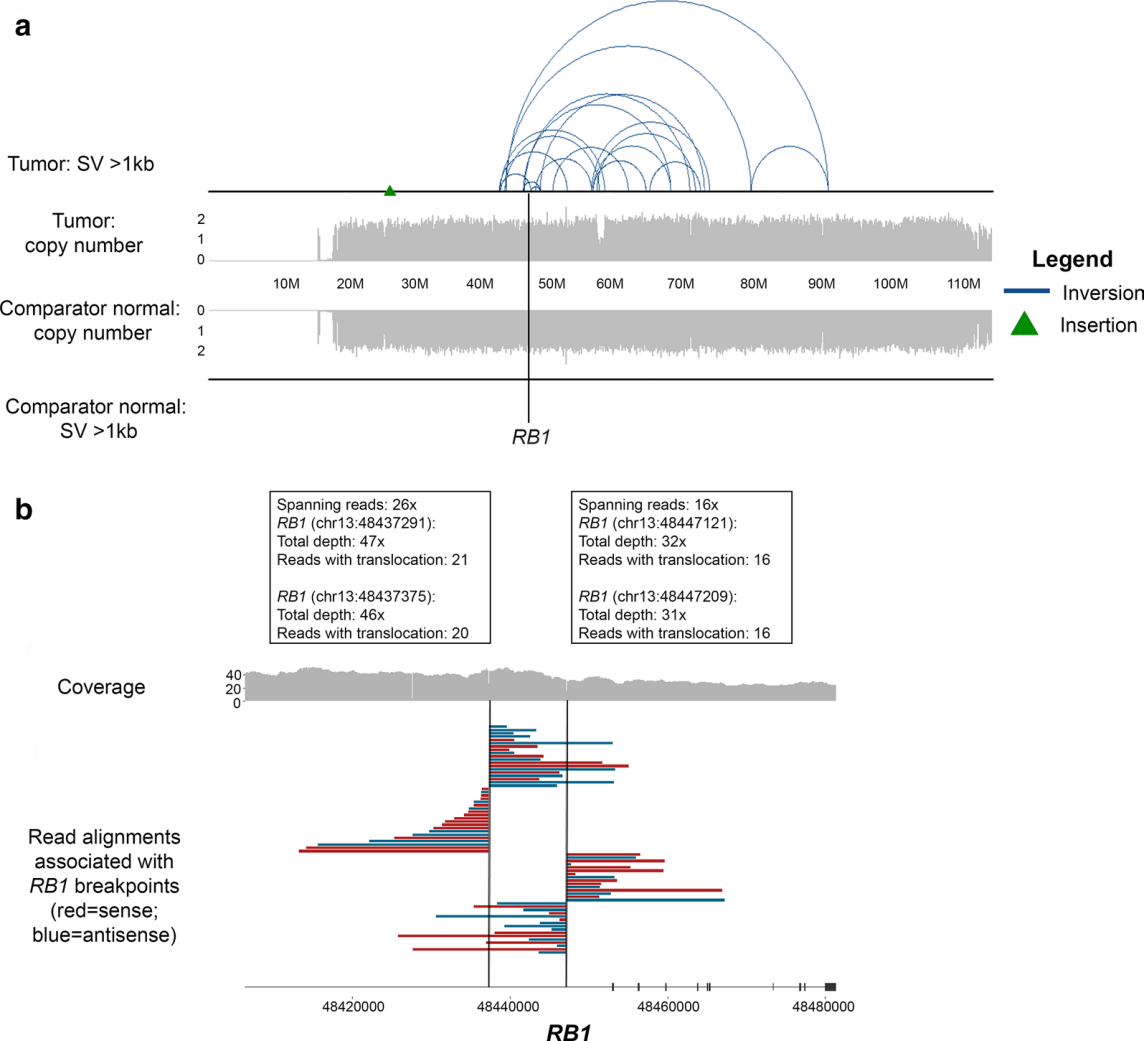
PacBio SMRT sequencing of paired tumor/normal DNA resolves *RB1* structural rearrangement. **a** Paired tumor/normal PacBio SMRT sequencing demonstrates complex structural variations (SVs) of the tumor aligned to GRCh38. Top: tumor SVs > 1 kb and copy number median-normalized to chromosome 13 coverage. Bottom: comparator normal SVs > 1 kb and copy number median-normalized to chromosome 13 coverage. Type of SVs denoted by a blue line (inversion) and green triangle (insertion). *RB1* is denoted by the vertical black line. **b** Two somatic rearrangements within intron 17 of *RB1*. Coverage calculated as average of 100 kb bins. Read alignments of breakpoint-associated reads in *RB1* are shown. Red reads are sense ( +) strand, blue reads are antisense (-) strand. Lines indicate regions of coverage depth loss corresponding to *RB1* SVs

Given the identification of multiple gene fusions near the *RB1* gene locus and the complete loss of RB1 protein expression by IHC, we utilized PacBio Single Molecule Real Time (SMRT) sequencing-based methodologies to resolve the genomic event. Evaluation of RNA by SMRT sequencing used the Iso-Seq method, providing reads that span entire transcripts to enable the characterization of full-length transcript isoforms. Iso-Seq sequencing of full-length transcript isoforms was utilized to elucidate the diversity of fusions in proximity to and encompassing the *RB1* locus. We confirmed the presence of both *RB1-SIAH3* (Fig. 4a, Additional File 1: Fig. S2) and *ZC3H13-KLHL1* in-frame fusion events (Additional File 1: Fig. S3a) by observing Iso-Seq reads with split alignments between the fusion partners. The *ZC3H13-KLHL1* chimeric protein is predicted to retain domains important for protein function, including the Broad-complex, Tramtrack, Bric-a-Brac/Poxvirus Zinc finger (BTB/POZ) domain and Kelch repeats (Additional File 1: Fig. S3b); however, the association of this fusion with tumorigenesis is uncertain [7]. The *RB1-SIAH3* fusion was noted to harbor putative aligned exon sequence derived from *SIAH3* intervening sequence. Furthermore, we identified a novel *RB1-*intergenic fusion aligning to a non-genic region of the genome (Fig. 4b). This fusion event presented as multiple potential isoforms, demonstrated by variability in putative aligned exon sequence supported by flanking splice donor and acceptor sequence. The predicted translation encodes a termination codon early within the putative aligned exon sequence; thus, we do not envisage a full-length chimeric protein to be produced. These findings lend support to a complex structural event that is unable to be fully resolved by NGS methodologies. To further resolve this event and predict the phase of the *RB1* fusion events, PacBio single-molecule HiFi circular consensus sequencing (CCS) of paired tumor/normal genomic DNA was performed [33]. The sequencing methodology generated highly accurate (> 99%) reads in excess of 10 kb. The HiFi reads were aligned to the human reference genome version 38 (GRCh38) using minimap2 and NGMLR, with structural variant calling performed with Sniffles, pbsv, and SVIM [10, 17, 28]. An ensemble-based approach was utilized to retain only SVs called by five different approaches. In the tumor tissue, predominately large inversion events spanning 13q14.11-q31.3 both involving and encompassing the *RB1* locus were identified (Fig. 5a, Additional File 1: Table S1).

On the basis of achieved read lengths of > 10 kb in HiFi sequencing data, we could predict the phase of certain somatic SVs in the tumor tissue. Importantly, we identified two somatic SVs hypothesized to occur in *trans* within intron 17 of the *RB1* gene based on the absence of reads spanning across both breakpoints (Fig. 5b, Additional File 1: Fig. S4). These events correspond to the *RB1-SIAH3* fusion and the *RB1-*non-genic rearrangement previously identified by next-generation and/or SMRT sequencing methodologies, indicating the complete absence of RB1 protein expression in the tumor is associated with somatic *RB1* loss-of-function SVs. Additionally, these data support the hypothesis that the driving event in this tumor is somatic in nature, rather than associated with germline *RB1* loss-of-function. Despite the complexity of SVs at this region, the copy number state of the chromosome is not altered; thus, it does not meet the criteria for chromothripsis [16] (Additional File 1: Fig. S5). Therefore, these events are a consequence of a larger, complex, copy-neutral structural rearrangement of chromosome 13q.

## Discussion and conclusions

Herein, we described a nine-month-old male with a sporadic, isolated, intracranial, sellar-suprasellar region retinoblastoma (i.e., without evidence of preceding, concomitant, or subsequent development of retinal or pineal gland neoplasia). DNA array-based methylation classified the tumor with high confidence as pineoblastoma group A/intracranial retinoblastoma.

Given the diffuse and strong staining essentially limited to synaptophysin, following performance of an extensive IHC stain panel, many CNS and extra-CNS small cell embryonal tumors were considered in our differential diagnosis. There was no C19MC locus amplification or Lin28a IHC reactivity, making embryonal tumor with multilayered rosettes (ETMR) or related tumor unlikely. Additionally, neither IHC nor FISH detected alterations in *BCOR*, *CIC*, or *NUTM1*, making newly described pediatric embryonal tumors such as the CNS high-grade neuroepithelial tumor with *BCOR* alteration and CNS Ewing sarcoma family tumor with *CIC* alteration unlikely. The absence of *EWSR1* rearrangement by FISH helped exclude intra- or extra-cranial Ewing’s sarcoma. Retained nuclear expression of INI-1 and Brg-1 excluded atypical teratoid/rhabdoid tumors. Comprehensive germ cell markers, including SALL4, were negative, ruling out poorly differentiated malignant germ cell tumor. Negative staining for Olig-2 and GFAP essentially ruled out a poorly differentiated high-grade glioma/astrocytoma. Negative staining for EMA and GFAP made a poorly differentiated ependymoma unlikely, and the negative Olig-2 made CNS neuroblastoma with FOXR2 activation unlikely. Although synaptophysin was positive, negative chromogranin staining made a poorly differentiated neuroendocrine tumor unlikely. The lack of glandular elements and lack of *DICER1* alteration made pituitary blastoma unlikely. Other negative IHC markers (e.g. myogenin, HMB-45, Neu-N, neurofilament, Oct 3/4, Glypican 3, CD1a, and Langerin) essentially excluded rhabdomyosarcoma, melanoma, malignant neuronal/glio-neuronal neoplasms, and Langerhans cell histiocytosis. Instead, the overall findings of diffuse synaptophysin reactivity, presence of Flexner-Wintersteiner-like rosettes, DNA array-based methylation classification, detected *RB1* alterations, and complete loss of RB1 expression by IHC in tumor cells, allowed a confident diagnosis of intracranial retinoblastoma.

Along with the tumor location, overall histopathology and molecular characteristics, the methylation-based classification allowed for an essentially definitive diagnosis of intracranial retinoblastoma in what would otherwise have been a difficult to classify embryonal neoplasm. Further molecular workup elucidated the mechanism of somatic *RB1* inactivation in this tumor in the absence of germline *RB1* or *DICER1* alterations. As part of a clinical trial for medulloblastomas and other CNS embryonal tumors, our patient was treated with intensive chemotherapy followed by three tandem stem-cell rescues and with no evidence of residual tumor at completion of therapy. Similar regimens have been described in the treatment of other intracranial retinoblastomas, obviating the use of radiotherapy [23].

The integration of classification by genome-wide DNA array-based methylation profiling into the clinical diagnostic paradigm for CNS tumors with challenging histopathology has aided in refining both diagnosis and clinical management [12]. The utility of this methodology in combination with comprehensive genomic profiling was evident in this patient, as standard histopathological work-up originally resulted in a less-definitive diagnosis of CNS embryonal neoplasm, NOS, for this infant.

Given the differential diagnoses of intracranial retinoblastoma and pituitary blastoma, potential germline alterations of *RB1* and *DICER1* were investigated but not found. Of clinical importance, children with hereditary *RB1* alterations are at increased risk for developing midline intracranial tumors, including pineal and sellar region tumors [11]. This phenomenon can be attributed to a common embryogenesis between the retinae and pineal gland, explaining the co-occurrence of retinoblastomas and pineoblastoma in patients with “trilateral” retinoblastoma. Moreover, the presence of ectopic photoreceptor cells along intracranial portions of the optic nerve system provides an explanation for the development of midline, intracranial, sellar region retinoblastoma [26, 27, 31]. Therefore, accurately identifying individuals with germline *RB1* alterations is critical for appropriate genetic counseling and clinical management (e.g., surveillance strategies and cancer risk assessment) [29]. Notably, diagnosis of intracranial retinoblastoma in an individual without hereditary retinoblastoma, incorporating DNA array-based methylation profiling, has previously been described [23], though this was in a patient with a concomitant pineal lesion. This report and the aforementioned prior study [23] highlight the utility of this methodology for this rare subset of non-heritable intracranial retinoblastoma.

Although the genomic landscape of retinoblastoma is diverse, large structural rearrangements are infrequently reported and, when described, are typically associated with chromothripsis [2, 15, 21, 22, 30]. Herein, the identification of the *RB1-SIAH3* and *ZC3H13-KLHL1* fusions by RNA-sequencing led us to further investigate the possibility of a larger chromosomal event. *RB1* and *SIAH3* reside in close proximity (~ 2 Mb apart) to one another on chromosome 13 but are transcribed in alternate directions. The chimeric protein product of the *RB1-SIAH3* fusion is predicted to truncate the linker domain and pocket domain B of RB1. The interaction of the RB1 pocket domains A and B with the linker domain is critical to tumor suppression and the E2F regulatory function associated with RB1 [9]. Through SMRT sequencing technologies, we identified a second *RB1* fusion with non-genic chromosome 13 sequence expected to disrupt RB1 protein expression. Both identified fusions demonstrated *RB1* breakpoints within intron 17. Although structural rearrangements in *RB1* are exceedingly rare, intron 17 breakpoints have been reported in multiple tumors with complex structural rearrangement in two independent studies, suggesting this may be a recurrent region for rearrangement [2, 20]. However, larger studies are necessary to confirm this finding. These fusions are predicted to result in complete loss-of-function, as evidenced by the absence of RB1 protein expression as demonstrated by IHC herein. Through multiple methodologies, we determined that the copy number state of chromosome 13 remained neutral, despite the presence of complex structural rearrangements.

Utilization of NGS methodologies to fully elucidate the complex biological mechanism of this intracranial retinoblastoma proved challenging. However, through the use of multiple sequencing methodologies and algorithms, we identified significant overlap in the high-confidence SVs. SMRT sequencing technologies overcome many of the limitations of NGS approaches which rely on short reads, including the ability to interrogate reads > 10 kb in length from a single DNA molecule for SV detection [19]. These approaches are more sensitive to larger chromosomal events than NGS technologies; the reads are more likely to encompass the breakpoint or the entire structural rearrangement and can provide the advantage of predicting phased genomic variation [19, 25]. Through SMRT sequencing methodologies, we were able to confirm two somatic rearrangements within *RB1*, predicted to be in* trans*, and supported by the absence of RB1 staining by IHC.

Our patient was treated on Head Start 4, a clinical trial for pediatric patients with medulloblastoma or other CNS embryonal tumors and randomized to undergo tandem autologous hematopoietic stem-cell rescues with three cycles of carboplatin and thiotepa. A prior report used a similar regimen of tandem high-dose chemotherapy with carboplatin, etoposide, cyclophosphamide, and thiotepa followed by autologous hematopoietic stem cell transplant for an infant with intracranial retinoblastoma [23]. In both our described patient and the prior case [23], a partial resection with a stable response was achieved, obviating the need for ancillary radiation therapy and its associated morbidity in this age group. Retrospective studies and case reports have shown the utilization of high-dose chemotherapy in the setting of trilateral retinoblastoma improves survival, although a standardized regimen has yet to be determined [4, 34, 35].

In summary, we report on an infant with an isolated, intracranial, sellar-suprasellar region retinoblastoma in the absence of a germline *RB1* alteration. A comprehensive approach to molecular profiling, including DNA array-based methylation profiling, Illumina NGS, and Pacific Bioscience SMRT sequencing, were critical for providing a definitive diagnosis and understanding the biological mechanism of *RB1* inactivation. The patient was treated using high-dose chemotherapy with autologous hematopoietic stem cell transplant, a regimen that has demonstrated benefit in small studies of trilateral retinoblastoma [4, 34, 35].

## Supplementary Information

**Additional file 1** Supplementary methods and data.

## Data Availability

The datasets generated during and/or analyzed during the current study are available in dbGAP (https://www.ncbi.nlm.nih.gov/gap/) [phs001820.v1.p1].

## Abbreviations

BCOR: BCL-6 interacting corepressor protein
*BCOR*: BCL-6 interacting corepressor gene
BRG1: Transcription activator BRG1
BTB/POZ: Broad-complex, Tramtrack, Bric-a-Brac/Poxvirus Zinc finger
CCS: Circular consensus sequencing
cDNA: Complimentary deoxyribonucleic acid
*CIC*: Capicua transcriptional repressor gene
CNA(s): Copy number alteration(s)
CNS: Central nervous system
*DICER1*: Dicer 1, ribonuclease III gene
*DLEU1*: Deleted In Lymphocytic Leukemia 1
*DNAJC12*: DnaJ Heat Shock Protein Family (Hsp40) Member C12 gene
dNTP(s): Deoxyribonucleotide triphosphate(s)
dsDNA: Double stranded deoxyribonucleic acid
DXA: Dual-energy X-ray absorptiometry
eES: Enhanced exome sequencing
EMA: Epithelial membrane antigen
ETMR: Embryonal tumor with multilayered rosettes
*EWSR1*: Ewing sarcoma breakpoint region 1 gene
FISH: Fluorescence in-situ hybridization
G-tube: Gastrostomy tube
GFAP: Glial fibrillary acid protein
GI: Gastrointestinal
GRCh37: Genome Reference Consortium Human Build 37
GRCh38: Genome Reference Consortium Human Build 38
GRCh38.p13: Genome Reference Consortium Human Build 38 patch release 13
GS: Genome sequencing
H&E: Hematoxylin and eosin
IHC: Immunohistochemical
IGM: The Steve and Cindy Rasmussen Institute for Genomic Medicine (IGM)
indel: Insertion-deletion
INI1: Integrase interactor 1
IRB: Institutional Review Board
*KLHL1*: Kelch-like 1 gene
LINE: Long interspersed nuclear element
LIN28: Lin-28 homolog A
LOH: Loss of heterozygosity
MRI: Magnetic resonance imaging
*MYCN*: N-MYC proto-oncogene
NCH: Nationwide Children’s Hospital
Neu-N: Neuronal nuclei protein
NGS: Next generation sequencing
NOS: Not otherwise specified
nt(s): Nucleotide(s)
NUTM1: Nuclear protein in testis family member 1 protein
PacBio: Pacific Biosciences
PBMCs: Peripheral blood mononuclear cells
PCR: Polymerase chain reaction
qPCR: Quantitative polymerase chain reaction
RB1: Retinoblastoma protein
*RB1*: RB Transcriptional Corepressor 1 gene
*RBP3*: Retinol Binding Protein 3 gene
RNA-seq: RNA-sequencing
RT-PCR: Reverse transcriptase polymerase chain reaction
*SIAH3*: Siah E3 Ubiquitin Protein Ligase Family Member 3 gene
SMRT: Single Molecule Real Time sequencing
SNV(s): Single nucleotide variant(s)
SV(s): Structural variation(s)
TPM: Transcripts per million
*TPT1*: Tumor Protein, Translationally-Controlled 1 gene
t-SNE: T-Distributed Stochastic Neighbor Embedding
*ZC3H13*: Zinc Finger CCCH-Type Containing 13 gene
