# Supplementary material for: Molecular classification of a complex structural rearrangement of the RB1 locus in an infant with sporadic, isolated, intracranial, sellar region retinoblastoma
Source: Acta Neuropathol Commun. 2021 Apr 7;9:61. doi: 10.1186/s40478-021-01164-z (PMC8025529; doi:10.1186/s40478-021-01164-z)
Supplement: Supplementary file 1 — Additional file 1 Supplementary methods and data. [file 40478_2021_1164_MOESM1_ESM.pdf]

Supplementary Information:

Molecular classification of a complex structural rearrangement of the *RB1* locus in an infant with sporadic, isolated, intracranial/sellar region retinoblastoma

Kathleen M. Schieffer, PhD[1], Alexander Z. Feldman, MD[2*], Esko A. Kautto, BS[1], Sean McGrath, MS[1], Anthony R. Miller, PhD[1], Maria Elena Hernandez-Gonzalez, MS[1], Stephanie LaHaye, PhD[1], Katherine E. Miller, PhD[1], Daniel C. Koboldt, MS[1,3], Patrick Brennan, MS[1], Benjamin Kelly, MS[1], Amy Wetzel, PhD[1], Vibhuti Agarwal, MD[4], Margaret Shatara, MD[5], Suzanne Conley, MS, CPNP-PC[6], Diana P. Rodriguez, MD[7], Rolla Abu-Arja, MD[6], Ala Shaikhkhalil, MD[8], Matija Snuderl, MD[9], Brent A. Orr, MD, PhD[10], Jonathan L. Finlay, MB ChB, FRCP, FRCPCH[6,11,12], Diana S. Osorio, MD[3,6,11], Annie I. Drapeau, MD[13,14], Jeffrey R. Leonard, MD[13,14], Christopher R. Pierson, MD, PhD[15,16,17], Peter White, PhD[1,3], Vincent Magrini, PhD[1,3], Elaine R. Mardis, PhD[1,3,14], Richard K. Wilson, PhD[1,3], Catherine E. Cottrell, PhD[1,3,16], Daniel R. Boué, MD, PhD[15,16]*

MATERIALS AND METHODS

Histology and immunohistochemistry

This tumor was reviewed by a board-certified pediatric pathologist and neuropathologist at Nationwide Children's Hospital (NCH) and outside consult performed by a board-certified neuropathologist at St. Jude Children's Research Hospital. At NCH, hematoxylin and eosin (H&E) sections from paraffin blocks were prepared to define representative tumor regions. Antibodies against the following antigens were applied: synaptophysin (27G12, Leica, cat#PA0299, monoclonal, prediluted), Ki-67 (SP6, Cell Marque, cat#275R-18, monoclonal, prediluted), Olig2 (Cell Marque, cat#387R-18, monoclonal [EP112], prediluted), Neu-N (Millipore Sigma, cat#MAB377, monoclonal, 1:200), neurofilament 200kD (Leica, PA0371, N52.1.7, prediluted), GFAP (Thermo [Neomarkers], cat#RB-087-R7, polyclonal, prediluted), p53 (Leica, cat#PA0057, monoclonal, prediluted), SALL4 (Abcam, cat#57577, monoclonal, 1:1600), EMA (Cell Marque, cat#247M-98, monoclonal [E29], prediluted), LIN28 (Cell Marque, cat#464R-18, monoclonal [EP150], prediluted), and INI1 (Cell Marque, cat#272M-18, monoclonal, prediluted) all run on a Leica Bond (III and/or MAX) Automated Immunostainer. Immunohistochemistry (IHC) for RB1 (BD Pharmingen, cat#554136, monoclonal [G3-245]) was performed by NeoGenomics. The following IHC staining and FISH analyses were performed by St. Jude Children's Research Hospital: BRG1 (Santa Cruz, cat# sc-17796, clone[G-7], 1:25), BCOR (Santa Cruz, cat# sc-514576, clone [C-10], 1:50), and NUTM1 (Cell Signaling, cat# 3625, clone[C52B1], 1:25) run on an automated immunostainers. Multicolor interphase FISH analysis for rearrangements of *BCOR* (Empire Genomics, Cat# BCORBA-20-GROR), *CIC* (Empire Genomics, Cat# CICBA-20-GROR), *EWSR1* (Abbott Molecular, Cat# 07J71-001, and

amplification of the C19MC locus at 19q13.42 (BACPAC Resources, BACS: RP11-984E8; CTD-2538G9; and CTD-2528A14) was performed.

DNA array methylation

Whole Genome DNA methylation analysis was performed at New York University Langone Health Molecular Pathology. DNA extraction was carried out from formalin-fixed paraffin embedded tissue using the automated clinical Maxwell system (Promega, Madison, WI). DNA methylation was analyzed using Illumina EPIC array as described previously [1, 18]. Molecular tumor classification was performed using clinically validated New York State approved random forest classifier as described previously [1].

DNA sequencing

Genome sequencing and enhanced exome sequencing was performed on DNA extracted from comparator peripheral blood mononuclear cells (PBMCs) and disease-involved snap frozen tumor. Libraries were prepared using 250 ng of input DNA beginning with enzymatic fragmentation followed by end repair, 5' phosphorylation, A-tailing, and sequencing adapter ligation using NEB Ultra II FS (New England Biolabs). Target enrichment by hybrid capture was performed with IDT xGen Exome Research Panel v1.0 enhanced with the xGenCNV Backbone Panel and Cancer spike-in (Integrated DNA Technologies, Coralville, IA). Paired-end 151-bp reads were generated on the Illumina HiSeq 4000. Secondary analysis was performed using Churchill, a comprehensive workflow for taking raw reads from alignment through to germline and somatic variants calls [8]. Reads were aligned to the human genome reference sequence (build GRCh37) using BWA (v0.7.15). Sequence alignments were refined according to

community-accepted guidelines for best practices (https://www.broadinstitute.org/gatk/guide/best-practices). Duplicate sequence reads were removed using samblaster-v.0.1.22, local realignment was performed on the aligned sequence data using the Genome Analysis Toolkit (v3.7–0), and Churchill's own deterministic implementation of base quality score recalibration was used. Germline variants were called using GATK's HaplotypeCaller. Enhanced exome sequencing (eES) average coverage depth was 283x and 287x for the tumor and normal sample, respectively. Genome sequencing average coverage depth was 119x and 41x for the tumor and normal sample, respectively. Somatic single nucleotide variation (SNV) and indel detection were performed using MuTect-2 [2]. Germline variation in cancer-associated genes and somatic variation across the coding region of the exome were analyzed [22]. Copy number alteration (CNA) was assessed using VarScan2 [10]. Genome sequencing data was analyzed for structural variation (SV) using LUMPY [11].

RNA-sequencing

In parallel, a 500 ng aliquot of snap frozen tumor derived RNA was subjected 0.8x SPRI bead cleanup and size selection prior to DNase treatment and ribodepletion prior to using Illumina's TruSeq Stranded Total RNA Sample preparation (performed with 8-minute chemical fragmentation). The library was constructed for whole transcriptome sequencing (RNA-seq). Paired-end 151-bp reads were generated on the Illumina HiSeq 4000, and reads were aligned to the human genome reference sequence (GRCh38) with the resultant output representing 94,103,657 uniquely mapped reads. RNA-seq data were processed using an ensemble approach of six fusion callers (STAR-fusion, JAFFA, fusioncatcher, FusionMap, MapSplice, and TopHat-Fusion) with high-confidence fusions characterized by overlap of fusion identification between

the multiple callers (called by at least 4 out of 6 callers) and rarity (<10% frequency) within our IGM Translational Research cancer cohort [3–5, 9, 13, 20, 21]. Transcript coverage was evaluated using GenVisR [19]. Transcripts per million (TPM) values were generated from paired-end RNA sequence data using Salmon with bootstrapping set to 100 [14].

Sanger sequencing

We used 500 ng of RNA with MultiScribe reverse transcriptase (ThermoFisher, Waltham, MA) and random hexamers (Applied Biosystems, Foster City, CA) for RT-PCR. Polymerase chain reaction (PCR) of complimentary DNA (cDNA) was performed with the forward primer in *RB1* exon 17 (5' TGATTCTGGAACAGATTTGTCTTT 3') and reverse primer in *SIAH3* exon 2 (5' CCTGGAGGATGTCAACCCTA 3'). PCR product was purified using the QIAquick purification kit (Qiagen, Germantown, MD). Forward and reverse Sanger sequencing reactions were performed with the Big Dye v3.1 terminator mix (ThermoFisher, Waltham, MA). Sequencing was performed on the Applied Biosystems 3130 and 3730 instruments.

PacBio HiFi SMRTbell template prep and sequencing

Genomic DNA quantity and quality, from primary tumor and blood comparator assayed with Thermo Fisher Scientific Qubit 1x dsDNA HS Assay Kit and Agilent TapeStation 4200 Genomic ScreenTape. DNA shearing was unnecessary as the DNA mode size for the primary tumor was 23.9 kb and blood comparator mode size was 13.6 kb which were in the size range for PacBio HiFi sample prep. HiFi SMRTbell libraries were prepare from 2.5 µg unsheared genomic DNA according to the PacBio HiFi Express Template Prep Kit 2.0 protocol (PN 101-853-100

Version 01 [September 2019]). Final libraries were diluted 1:5 with Buffer EB and assessed for DNA concentration by Qubit 1x dsDNA HS and size by TapeStation 4200 Genomic ScreenTape.

Library metrics metadata values including available volume, concentration, and average insert size were entered into the SMRT Link Sample Setup worksheet. The worksheet calculates the required SMRTbell template, sequencing primer and polymerase for each library. For HiFi, SMRTbell libraries were complexed with Sequel II Bind Kit 2.0 to "on plate" concentration of 55 pM. The primary tumor was sequenced to 30x and the blood comparator was sequenced to 15x mapped coverage on the Sequel II instrument with Sequel II Sequencing Kit 2.0 chemistry, 4-hour pre-extension plus 30-hour movie time and Predictive Loading per cell. Data from all primary tumor or blood comparator SMRT cells were analyzed with SMRT Link "CCS with Mapping" analysis application (SMRT Link Version 8.0.0.79519, hg38 reference, maximum CCS read length = 50,000, minimum CCS read length = 10, minimum number of passes = 3, minimum predicted accuracy = 0.99).

PacBio HiFi-based analysis of chromosome 13

SVs of >1 kb were identified from the HiFi Circular Consensus Sequencing (CCS) data using an ensemble-based approach. Briefly, we used five methods to call SVs from the data: reads aligned with NGMLR [17] (version 0.2.7) and called with Sniffles [17] (version 1.0.11) and SVIM [6] (version 1.4.0); reads aligned with minimap2 [12] (version 2.17-r941) and called with Sniffles and SVIM; and reads aligned with pbmm2 (version 1.1.0) and called with pbsv (version 2.2.2). In all cases, reads were aligned to the GRCh38 patch release 13 (GRCh38.p13) using preset parameters for PacBio and/or CCS data.

Our ensemble approach collected filter passing variants from each call set and compared them for compatible structural variant types and coordinates, allowing for a 50 nucleotide (nt) margin to detect overlaps. Only SVs which were present in all five call sets, affected a region of 1000 nts or above, and had a minimum of 4 supporting reads were accepted into the final ensemble-based output. The algorithm Sniffles flags variants with read strand bias which were additionally filtered out during our approach.

We focused our analysis on chromosome 13, as other assays suggested the presence of significant rearrangements. Our method detected 21 large deletions and 5 large insertions (>1 kb) in the comparator normal sample. The tumor sample had 22 large deletions, 8 large insertions, and 26 inversion-associated rearrangements. One inversion was additionally detected in the comparator normal sample, but further analysis revealed it to be an artifact of incorrect alignment due to overlap with a long interspersed nuclear element (LINE). A filtering strategy was employed to eliminate systematic false positives and exclude variants that represent normal genetic variation in the human genome. SVs were compared to five normal human cell lines and 15 non-cancer samples from institutional patients. A frequency-based filtering was used to exclude recurrent variants found at >20% of this internal reference cohort. Additionally, SVs were filtered for normal human population variation through comparison against gnomAD SV v2.1 (<0.001 frequency) [7]. All of the SVs found in the comparator normal sample were considered normal genetic variation within the human genome.

Copy number estimations were based on median-normalized coverage data. Bedtools [16] (version 2.29.2) genomecov was used to generate alignment-based coverage, which was then grouped into 100 kb blocks. Median normalized coverage was calculated as

(*coverage/median(coverage))*. The log2-based copy number was calculated as *2*log2([median-norm tumor coverage]/[median-norm normal coverage])*.

Small variants (SNVs and small indels) were called from the NGMLR-aligned data using Google DeepVariant (version 0.9.0) [15].

Iso-Seq SMRTbell Sample Prep and Sequencing

NEBNext Single Cell/Low Input cDNA Synthesis

Total RNA was subjected to DNase treatment (Zymo RNA Clean and Concentrator-5) according to manufacturer's protocol. Briefly, 300 ng of treated total RNA was reverse transcribed using NEBNext Single Cell/Low Input kit for cDNA synthesis following the steps outlined within Iso-Seq Express Template Procedure & Checklist (PN 101-763-800 Version 02 [October 2019]) in keeping with protocol recommendations with the following specified exceptions. Resulting cDNA was purified and size selected at >1.0 kb utilizing 1.0x ProNex Beads (Promega) with final elution being 17 µl in Buffer EB (Qiagen). PCR amplification was performed using Takara PrimeStar GXL (2.50 Units total) with NEBNext Single Cell (catalog #E6421S) and ISO-Seq Express (PN #101-737-500) cDNA primers added during first-strand cDNA synthesis. Optimal PCR cycling was derived by real-time PCR using KAPA SYBR FAST quantitative PCR (qPCR) Master Mix, with Single Cell and ISO-Seq Express cDNA PCR Primers in a 50 µl reaction containing 1 µl template run on AriaMX Real-Time PCR System (Agilent) with the following conditions (5 minute denaturation at 95ºC, 35 cycles of 10 seconds at 95ºC, 30 second extension at 65ºC, 5 minutes at 72ºC). Number of PCR amplification cycles is determinate upon the mid-point of the exponential phase within the amplification curve. Sample cDNA amplification occurred in duplicate 50 µl PCR reactions with each containing 8 µl

purified first-strand cDNA template, 1x PrimeSTAR GXL Buffer, 0.1 mM deoxyribonucleotide triphosphates (dNTPs), 1.25 Units PrimeSTAR GXL DNA Polymerase (1.25U/µl), NEBNext Single Cell and ISO-Seq Express cDNA PCR Primers. PCR parameters were the following: 30 second denaturation at 98ºC, 17 cycles of 10 seconds at 98ºC, 15 seconds at 65ºC, 10 minutes at 68ºC, and final extension of 5 minutes at 68ºC.

cDNA Size Fractionation

PCR reactions were combined after amplification and size selected at >2 kb with 0.4x SPRIselect paramagnetic beads (Beckman Coulter). Resulting supernatant was transferred to a fresh tube containing additional 20 µl SPRIselect beads yielding a double size-selected product (0.4/0.6x) between 0.5 – 2 kb. Bead associated cDNA was eluted and concentrated in 12 µl Buffer EB. Fractionated second-strand cDNA was pooled for a final yield of 500 ng at a 1:1 molar ratio of >2 kb (0.4x) and 0.5 – 2 kb (0.4/0.6x) sized cDNA for PacBio Iso-Seq and preferential sequencing of larger sized molecules.

SMRTbell Template Prep and Sequel II Sequencing

SMRTbell library was prepared using SMRTbell Express Template Prep Kit 2.0 according to PacBio Iso-Seq protocol (PN 101-763-800 Version 02 [October 2019]). A five-fold dilution derived from final SMRTbell library was used for assessing concentration and average library size by Qubit double stranded (dsDNA) HS and 2100 Bioanalyzer High Sensitivity DNA Kit (Agilent), respectively. Volumetric calculations were done on a per sample basis using SMRT Link Sample Setup for v4 primer and polymerase binding conditions dependent upon initial SMRTbell library molarity. The SMRTbell library was complexed with Sequel II Binding

Kit 2.0, purified with 1.0x ProNex beads and sequenced at 50 pM on-plate loading concentration. Sample was sequenced on a single 8M SMRT Cell on the PacBio Sequel II platform using v2.0EA chemistry and 2-hour pre-extension followed by 24-hour movie collection.

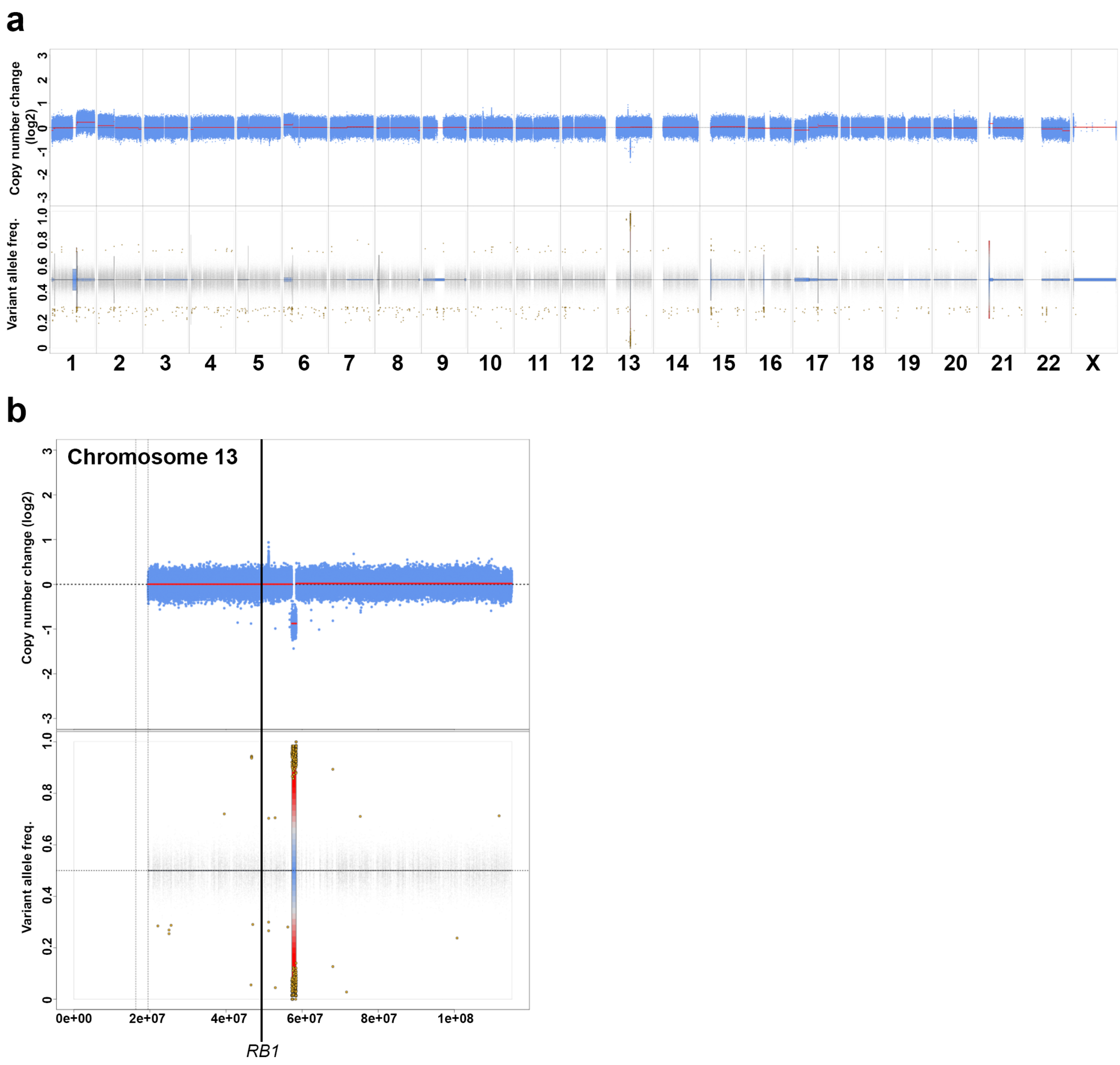

Fig. S1: Whole genome sequencing copy number variation. (**a**) Genome-wide somatic copy number alterations (CNAs) are shown. (**a**) Top: tumor CNAs relative to matched normal in log2 scale. Blue points represent log2 values based on sequence depth in 100-bp windows. Red lines indicate segmented CNA calls. Bottom: tumor variant allele frequency for variants that are non-reference calls in the normal. Points in red indicate significant loss of heterozygosity (LOH). The x-axis denotes the chromosome number. (**b**) Same as in (**a**) but for chromosome 13. *RB1* is denoted by the vertical black line.

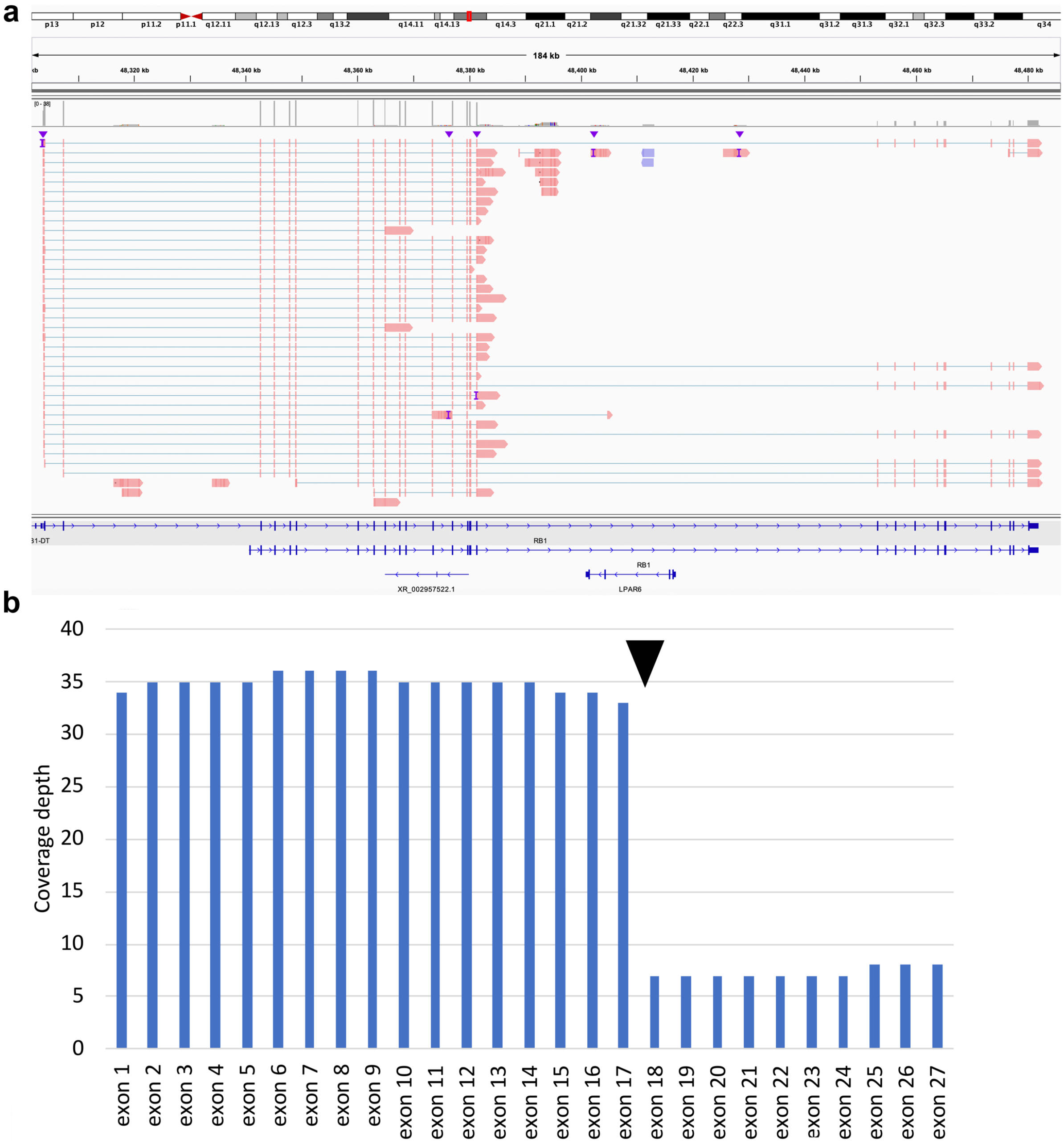

Fig. S2: Iso-Seq visualization of *RB1* locus. **(a)** Integrated Genomics Viewer (IGV) screenshot of *RB1* transcripts aligned to GRCh38 associated with complex structural rearrangements truncate at exon 17 or full-length *RB1* transcript (NM_000321). **(b)** Exon-level coverage from Iso-Seq demonstrates a loss of coverage depth following the fusion breakpoints in intron 17. Low-level full-length *RB1* transcript was also noted in RNA-sequencing data and likely corresponds to admixed normal brain tissue. The arrowhead denotes the fusion breakpoint.

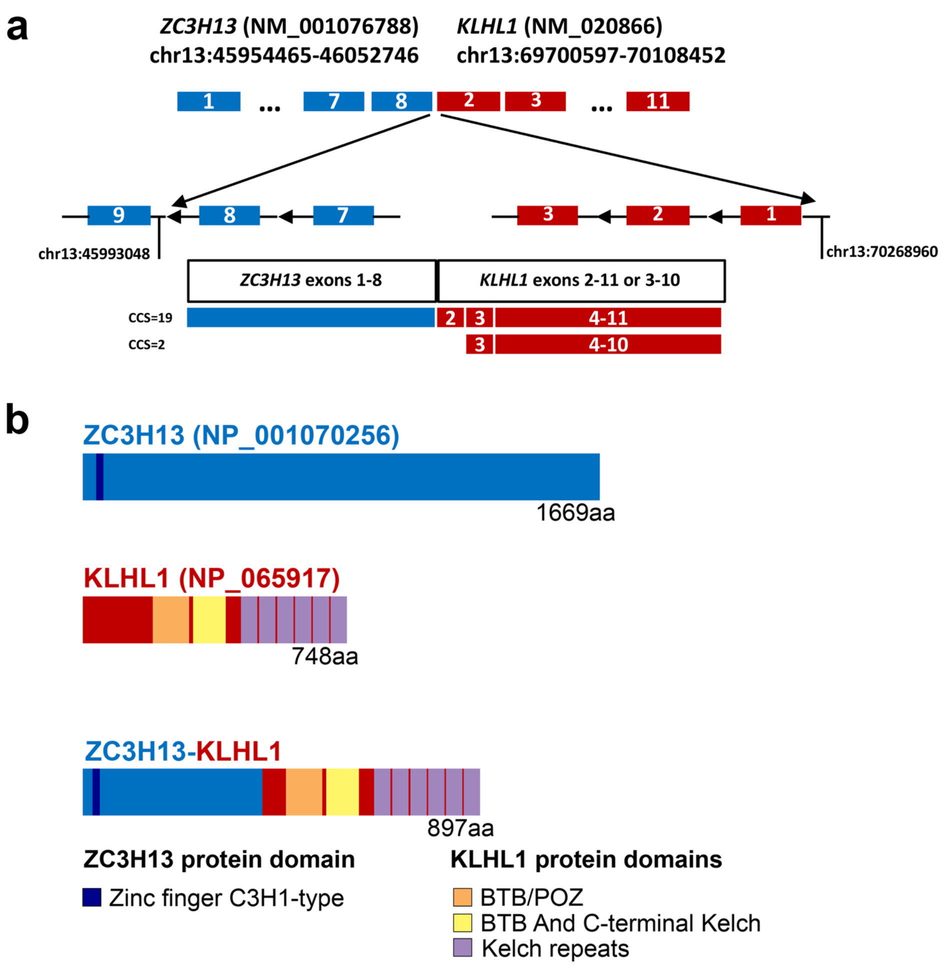

Fig. S3: In-frame intergenic fusion of *ZC3H13-KLHL1*. **(a)** PacBio Iso-Seq *ZC3H13-KLHL1* transcript isoform structure and diversity. SMRT sequencing of tumor-derived RNA using the Iso-Seq method aligned to GRCh38. Multiple transcript isoforms of the in-frame *ZC3H13-KLHL1* fusion are shown. The supporting circular consensus reads (CCSs) are shown for each isoform. **(b)** Protein structure of the *ZC3H13-KLHL1* fusion. BTB/POZ, Broad-complex, Tramtrack, Bric-a-Brac/Poxvirus Zinc finger

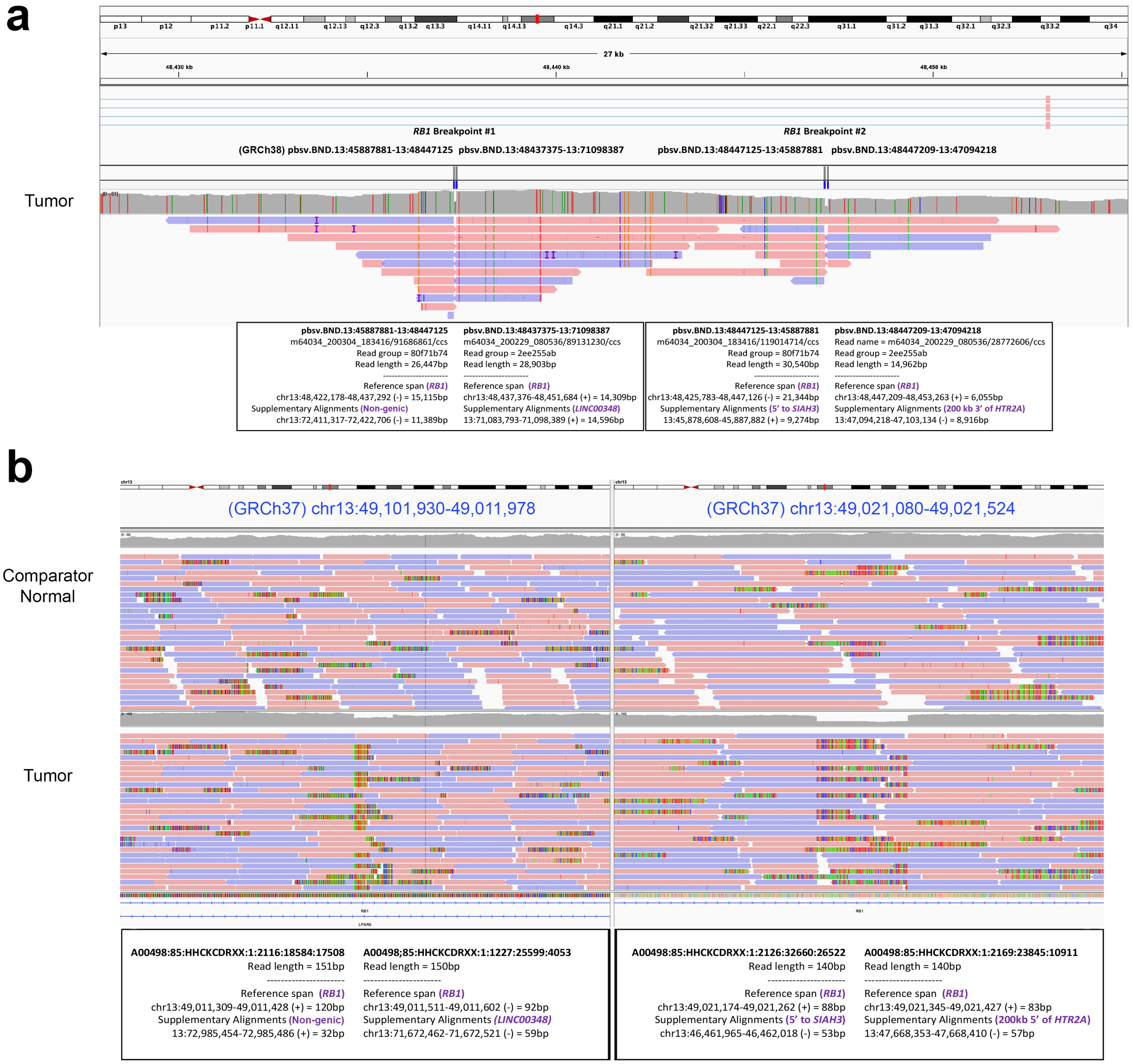

Fig. S4: *RB1* (NM_000321) intron 17 harbors two large structural variants predicted in *trans*. **(a)** Integrated Genomics Viewer (IGV) screenshot of two structural variants in intron 17 identified by Single Molecule Real Time (SMRT) HiFi circular consensus sequencing of tumor DNA (aligned to human reference genome GRCh38). Given the absence of long read sequences spanning both breakpoints, the events are hypothesized to be in *trans*. **(b)** Illumina genome sequencing demonstrating the same complex rearrangement with supplementary read alignments corresponding to the *RB1-SIAH3* and *RB1*-non-genic fusions (aligned to human reference genome GRCh37). The comparator normal and tumor are shown with reads corresponding to the structural variants only identified in the tumor.

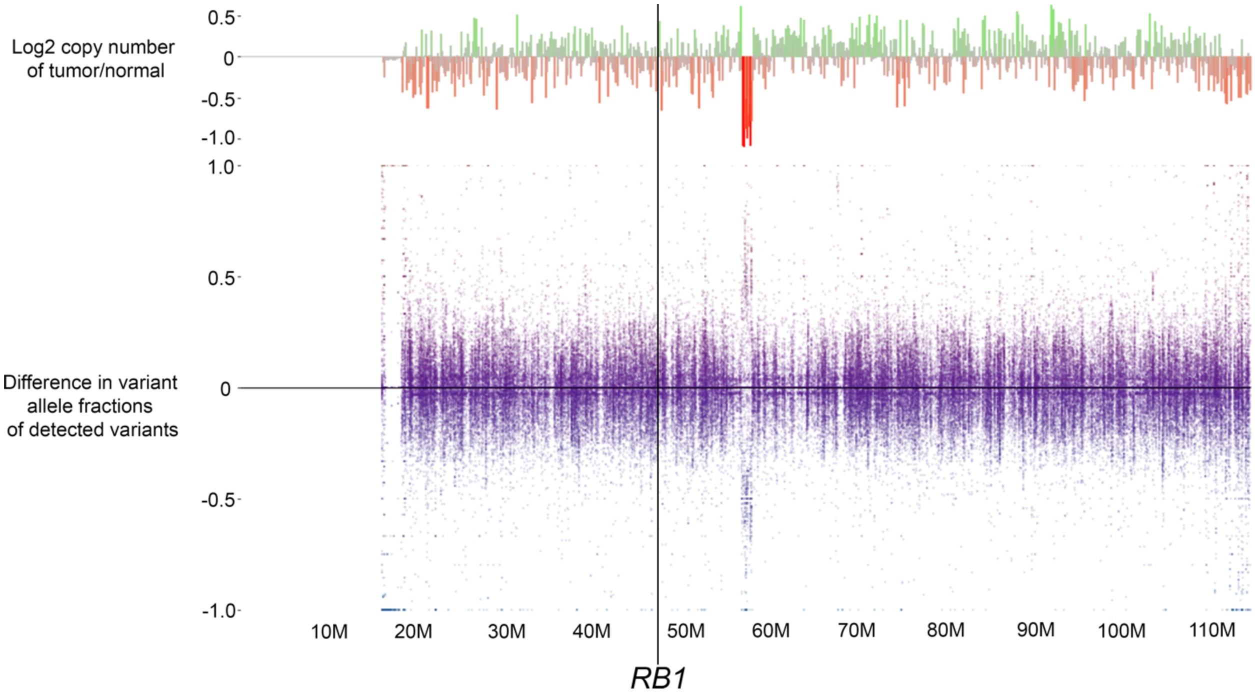

Fig. S5: Paired tumor/normal copy number analysis derived from PacBio SMRT sequencing from DNA. Top: log2 copy number of tumor/normal ratio of chromosome 13, using 100 kb bins. Bottom: difference in variant allele fractions of detected non-reference variants. Values > 0 indicate that the variant is detected at a higher fraction in the tumor; values < 0 indicate that variant is detected at a higher fraction in the comparator normal sample. *RB1* is denoted by the black vertical line.

Table S1: Structural Variation from Paired Tumor-Normal HiFi Long Read Sequencing

Genomic coordinates (GRCh38)	Type of structural variant	Protein-coding genes	Read support (Minimap2 w/Sniffles)	Read support (Ensemble mode)	LUMPY PE (Illumina genome sequencing)	LUMPY SR (Illumina genome sequencing)
chr13:26472812-26475067	insertion	none	10	7	ND	ND
chr13:42472185-52379638	inversion[1]	*TNFSF11, FAM216B, EPSTI1, DNAJC15, ENOX1, CCDC122, LACC1, SMIM2, SERP2, TSC22D1, NUFIP1, GPALPP1, GTF2F2, KCTD4, TPT1, SLC25A30, COG3, ERICH6B, CBY2, SIAH3, ZC3H13, CPB2, LCP1, LRRC63, RUBCNL, LRCH1, ESD, HTR2A, SUCLA2, NUDT15, MED4, ITM2B,* ***RB1****, LPAR6, RCBTB2, CYSLTR2, FNDC3A, MLNR, CDADC1, CAB39L, SETDB2, PHF11, RCBTB1, ARL11, EBPL, KPNA3, SPRYD7, TRIM13, KCNRG, DLEU7, RNASEH2B, C13ORF42, FAM124A, SERPINE3, INTS6, WDFY2, DHRS12, TMEM272, CCDC70, ATP7B, ALG11, UTP14C, NEK5, NEK3, THSD1*	9	9	51	18
chr13:42603431-47093292	inversion[1]	*TNFSF11, FAM216B, EPSTI1, DNAJC15, ENOX1, CCDC122, LACC1, SMIM2, SERP2, TSC22D1, NUFIP1, GPALPP1, GTF2F2, KCTD4, TPT1, SLC25A30, COG3, ERICH6B, CBY2, SIAH3, ZC3H13, CPB2, LCP1, LRRC63, RUBCNL, LRCH1, ESD, HTR2A*	21	21	36	33
chr13:42603514-57872641	inversion[2]	*TNFSF11, FAM216B, EPSTI1, DNAJC15, ENOX1, CCDC122, LACC1, SMIM2, SERP2, TSC22D1, NUFIP1, GPALPP1, GTF2F2, KCTD4, TPT1, SLC25A30, COG3, ERICH6B, CBY2, SIAH3, ZC3H13, CPB2, LCP1, LRRC63, RUBCNL, LRCH1, ESD, HTR2A, SUCLA2, NUDT15, MED4, ITM2B,* ***RB1****, LPAR6, RCBTB2, CYSLTR2, FNDC3A, MLNR, CDADC1, CAB39L, SETDB2, PHF11, RCBTB1, ARL11, EBPL, KPNA3, SPRYD7, TRIM13, KCNRG, DLEU7, RNASEH2B, C13ORF42, FAM124A, SERPINE3, INTS6, WDFY2, DHRS12, TMEM272, CCDC70, ATP7B, ALG11, UTP14C, NEK5, NEK3, THSD1, VPS36, CKAP2, HNRNPA1L2, SUGT1, CNMD, PCDH8, OLFM4, PRR20A, PRR20C, PRR20B, PRR20D, PRR20E, PCDH17*	15	14	9	0

chr13:43315339-57076504	inversion[3]	*ENOX1, CCDC122, LACC1, SMIM2, SERP2, TSC22D1, NUFIP1, GPALPP1, GTF2F2, KCTD4, TPT1, SLC25A30, COG3, ERICH6B, CBY2, SIAH3, ZC3H13, CPB2, LCP1, LRRC63, RUBCNL, LRCH1, ESD, HTR2A, SUCLA2, NUDT15, MED4, ITM2B,* ***RB1****, LPAR6, RCBTB2, CYSLTR2, FNDC3A, MLNR, CDADC1, CAB39L, SETDB2, PHF11, RCBTB1, ARL11, EBPL, KPNA3, SPRYD7, TRIM13, KCNRG, DLEU7, RNASEH2B, C13ORF42, FAM124A, SERPINE3, INTS6, WDFY2, DHRS12, TMEM272, CCDC70, ATP7B, ALG11, UTP14C, NEK5, NEK3, THSD1, VPS36, CKAP2, HNRNPA1L2, SUGT1, CNMD, PCDH8, OLFM4*	17	17	34	50
chr13:43316396-79221731	inversion[1]	*ENOX1, CCDC122, LACC1, SMIM2, SERP2, TSC22D1, NUFIP1, GPALPP1, GTF2F2, KCTD4, TPT1, SLC25A30, COG3, ERICH6B, CBY2, SIAH3, ZC3H13, CPB2, LCP1, LRRC63, RUBCNL, LRCH1, ESD, HTR2A, SUCLA2, NUDT15, MED4, ITM2B,* ***RB1****, LPAR6, RCBTB2, CYSLTR2, FNDC3A, MLNR, CDADC1, CAB39L, SETDB2, PHF11, RCBTB1, ARL11, EBPL, KPNA3, SPRYD7, TRIM13, KCNRG, DLEU7, RNASEH2B, C13ORF42, FAM124A, SERPINE3, INTS6, WDFY2, DHRS12, TMEM272, CCDC70, ATP7B, ALG11, UTP14C, NEK5, NEK3, THSD1, VPS36, CKAP2, HNRNPA1L2, SUGT1, CNMD, PCDH8, OLFM4, PRR20A, PRR20C, PRR20B, PRR20D, PRR20E, PCDH17, DIAPH3, TDRD3, PCDH20, PCDH9, KLHL1, DACH1, MZT1, BORA, DIS3, PIBF1, KLF5, KLF12, TBC1D4, COMMD6, UCHL3, LMO7, KCTD12, ACOD1, CLN5, FBXL3, MYCBP2, SCEL, SLAIN1, EDNRB, POU4F1, OBI1*	17	17	27	36
chr13:43465232-90486512	inversion[3]	*ENOX1, CCDC122, LACC1, SMIM2, SERP2, TSC22D1, NUFIP1, GPALPP1, GTF2F2, KCTD4, TPT1, SLC25A30, COG3, ERICH6B, CBY2, SIAH3, ZC3H13, CPB2, LCP1, LRRC63, RUBCNL, LRCH1, ESD, HTR2A, SUCLA2, NUDT15, MED4, ITM2B,* ***RB1****, LPAR6, RCBTB2, CYSLTR2, FNDC3A, MLNR, CDADC1, CAB39L, SETDB2, PHF11, RCBTB1, ARL11, EBPL, KPNA3, SPRYD7, TRIM13, KCNRG, DLEU7, RNASEH2B, C13ORF42, FAM124A, SERPINE3, INTS6, WDFY2, DHRS12,*	18	17	30	46

		TMEM272, CCDC70, ATP7B, ALG11, UTP14C, NEK5, NEK3, THSD1, VPS36, CKAP2, HNRNPA1L2, SUGT1, CNMD, PCDH8, OLFM4, PRR20A, PRR20C, PRR20B, PRR20D, PRR20E, PCDH17, DIAPH3, TDRD3, PCDH20, PCDH9, KLHL1, DACH1, MZT1, BORA, DIS3, PIBF1, KLF5, KLF12, TBC1D4, COMMD6, UCHL3, LMO7, KCTD12, ACOD1, CLN5, FBXL3, MYCBP2, SCEL, SLAIN1, EDNRB, POU4F1, OBI1, RBM26, NDFIP2, SPRY2, SLITRK1, SLITRK6, SLITRK5				
chr13:45887881-48447125	inversion[1]	*ZC3H13, CPB2, LCP1, LRRC63, RUBCNL, LRCH1, ESD, HTR2A, SUCLA2, NUDT15, MED4, ITM2B,* ***RB1****, LPAR6*	16	16	25	31
chr13:45991409-67505672	inversion[1]	*ZC3H13, CPB2, LCP1, LRRC63, RUBCNL, LRCH1, ESD, HTR2A, SUCLA2, NUDT15, MED4, ITM2B,* ***RB1****, LPAR6, RCBTB2, CYSLTR2, FNDC3A, MLNR, CDADC1, CAB39L, SETDB2, PHF11, RCBTB1, ARL11, EBPL, KPNA3, SPRYD7, TRIM13, KCNRG, DLEU7, RNASEH2B, C13ORF42, FAM124A, SERPINE3, INTS6, WDFY2, DHRS12, TMEM272, CCDC70, ATP7B, ALG11, UTP14C, NEK5, NEK3, THSD1, VPS36, CKAP2, HNRNPA1L2, SUGT1, CNMD, PCDH8, OLFM4, PRR20A, PRR20C, PRR20B, PRR20D, PRR20E, PCDH17, DIAPH3, TDRD3, PCDH20, PCDH9*	20	19	29	38
chr13:46140147-70269767	inversion[3]	*LCP1, LRRC63, RUBCNL, LRCH1, ESD, HTR2A, SUCLA2, NUDT15, MED4, ITM2B,* ***RB1****, LPAR6, RCBTB2, CYSLTR2, FNDC3A, MLNR, CDADC1, CAB39L, SETDB2, PHF11, RCBTB1, ARL11, EBPL, KPNA3, SPRYD7, TRIM13, KCNRG, DLEU7, RNASEH2B, C13ORF42, FAM124A, SERPINE3, INTS6, WDFY2, DHRS12, TMEM272, CCDC70, ATP7B, ALG11, UTP14C, NEK5, NEK3, THSD1, VPS36, CKAP2, HNRNPA1L2, SUGT1, CNMD, PCDH8, OLFM4, PRR20A, PRR20C, PRR20B, PRR20D, PRR20E, PCDH17, DIAPH3, TDRD3, PCDH20, PCDH9, KLHL1*	19	18	21	42
chr13:47094218-48447208	inversion[1]	*SUCLA2, NUDT15, MED4, ITM2B,* ***RB1****, LPAR6*	15	15	19	43
chr13:47756050-55967810	inversion[1]	*SUCLA2, NUDT15, MED4, ITM2B,* ***RB1****, LPAR6, RCBTB2, CYSLTR2, FNDC3A, MLNR, CDADC1, CAB39L, SETDB2, PHF11, RCBTB1, ARL11, EBPL, KPNA3,*	8	8	41	36

		SPRYD7, TRIM13, KCNRG, DLEU7, RNASEH2B, C13ORF42, FAM124A, SERPINE3, INTS6, WDFY2, DHRS12, TMEM272, CCDC70, ATP7B, ALG11, UTP14C, NEK5, NEK3, THSD1, VPS36, CKAP2, HNRNPA1L2, SUGT1, CNMD, PCDH8, OLFM4				
chr13:48437291-72411315	inversion[3]	***RB1**, LPAR6, RCBTB2, CYSLTR2, FNDC3A, MLNR, CDADC1, CAB39L, SETDB2, PHF11, RCBTB1, ARL11, EBPL, KPNA3, SPRYD7, TRIM13, KCNRG, DLEU7, RNASEH2B, C13ORF42, FAM124A, SERPINE3, INTS6, WDFY2, DHRS12, TMEM272, CCDC70, ATP7B, ALG11, UTP14C, NEK5, NEK3, THSD1, VPS36, CKAP2, HNRNPA1L2, SUGT1, CNMD, PCDH8, OLFM4, PRR20A, PRR20C, PRR20B, PRR20D, PRR20E, PCDH17, DIAPH3, TDRD3, PCDH20, PCDH9, KLHL1, DACH1*	21	20	23	33
chr13:50286147-61304029	inversion[1]	*DLEU7, RNASEH2B, C13ORF42, FAM124A, SERPINE3, INTS6, WDFY2, DHRS12, TMEM272, CCDC70, ATP7B, ALG11, UTP14C, NEK5, NEK3, THSD1, VPS36, CKAP2, HNRNPA1L2, SUGT1, CNMD, PCDH8, OLFM4, PRR20A, PRR20C, PRR20B, PRR20D, PRR20E, PCDH17, DIAPH3, TDRD3*	19	18	32	22
chr13:55968403-73180366	inversion[1]	*PRR20A, PRR20C, PRR20B, PRR20D, PRR20E, PCDH17, DIAPH3, TDRD3, PCDH20, PCDH9, KLHL1, DACH1, MZT1, BORA, DIS3, PIBF1, KLF5*	22	22	29	28
chr13:56149426-63887928	inversion[3]	*PRR20A, PRR20C, PRR20B, PRR20D, PRR20E, PCDH17, DIAPH3, TDRD3, PCDH20*	23	23	27	45
chr13:56152756-67507608	inversion[1]	*PRR20A, PRR20C, PRR20B, PRR20D, PRR20E, PCDH17, DIAPH3, TDRD3, PCDH20, PCDH9*	25	24	31	36
chr13:56711745-71099680	inversion[3]	*PRR20A, PRR20C, PRR20B, PRR20D, PRR20E, PCDH17, DIAPH3, TDRD3, PCDH20, PCDH9, KLHL1*	15	14	27	44
chr13:64437931-71849306	inversion[1]	*PCDH9, KLHL1, DACH1*	21	20	24	33
chr13:79221513-90486137	inversion[1]	*RBM26, NDFIP2, SPRY2, SLITRK1, SLITRK6, SLITRK5*	18	17	33	35

ND, not detected; PE, paired-end reads; SR, split reads. Using genome sequencing (GS), the type of structural variation differed than SMRT sequencing: [1]breakend with only a single genomic coordinate, [2]duplication, [3]deletion. All read support counts are derived from the disease-involved tissue. No reads were identified in the comparator normal sample by either methodology.

REFERENCES

1. Capper D, Jones DTW, Sill M, Hovestadt V, Schrimpf D, Sturm D, *et al.* (2018) DNA methylation-based classification of central nervous system tumours. Nature 555:469–474. doi: 10.1038/nature26000
2. Cibulskis K, Lawrence MS, Carter SL, Sivachenko A, Jaffe D, Sougnez C, *et al.* (2013) Sensitive detection of somatic point mutations in impure and heterogeneous cancer samples. Nat Biotechnol 31:213–219. doi: 10.1038/nbt.2514
3. Davidson NM, Majewski IJ, Oshlack A (2015) JAFFA: High sensitivity transcriptome-focused fusion gene detection. Genome Med 7:43. doi: 10.1186/s13073-015-0167-x
4. Ge H, Liu K, Juan T, Newman M, Hoeck W, Bateman A (2011) FusionMap: detecting fusion genes from next-generation sequencing data at base-pair resolution. Bioinformatics 27:1922–1928. doi: 10.1093/bioinformatics/btr310
5. Haas BJ, Dobin A, Li B, Stransky N, Pochet N, Regev A (2019) Accuracy assessment of fusion transcript detection via read-mapping and de novo fusion transcript assembly-based methods. Genome Biol 20:1–16. doi: 10.1186/s13059-019-1842-9
6. Heller D, Vingron M (2019) SVIM: structural variant identification using mapped long reads. Bioinformatics 35:2907–2915
7. Karczewski KJ, Francioli LC, Tiao G, Cummings BB, Alföldi J, Wang Q, *et al.* (2020) The mutational constraint spectrum quantified from variation in 141,456 humans. Nature 581:434–443. doi: 10.1038/s41586-020-2308-7
8. Kelly BJ, Fitch JR, Hu Y, Corsmeier DJ, Zhong H, Wetzel AN, *et al.* (2015) Churchill: An ultra-fast, deterministic, highly scalable and balanced parallelization strategy for the discovery of human genetic variation in clinical and population-scale genomics. Genome

Biol 16:6. doi: 10.1186/s13059-014-0577-x

9. Kim D, Salzberg SL (2011) TopHat-Fusion: An algorithm for discovery of novel fusion transcripts. Genome Biol 12:R72. doi: 10.1186/gb-2011-12-8-r72

10. Koboldt DC, Zhang Q, Larson DE, Shen D, McLellan MD, Lin L, *et al.* (2012) VarScan 2: Somatic mutation and copy number alteration discovery in cancer by exome sequencing. Genome Res 22:568–576. doi: 10.1101/gr.129684.111

11. Layer RM, Chiang C, Quinlan AR, Hall IM (2014) LUMPY: A probabilistic framework for structural variant discovery. Genome Biol 15:R84. doi: 10.1186/gb-2014-15-6-r84

12. Li H (2018) Minimap2: pairwise alignment for nucleotide sequences. Bioinformatics 34:3094–3100

13. Nicorici D, Satalan M, Edgren H, Kangaspeska S, Murumagi A, Kallioniemi O, *et al.* (2014) FusionCatcher - a tool for finding somatic fusion genes in paired-end RNA-sequencing data. Cold Spring Harbor Labs Journals

14. Patro R, Duggal G, Love MI, Irizarry RA, Kingsford C (2017) Salmon provides fast and bias-aware quantification of transcript expression. Nat Methods 14:417–419. doi: 10.1038/nmeth.4197

15. Poplin R, Chang PC, Alexander D, Schwartz S, Colthurst T, Ku A, *et al.* (2018) A universal snp and small-indel variant caller using deep neural networks. Nat Biotechnol 36:983. doi: 10.1038/nbt.4235

16. Quinlan AR, Hall IM (2010) BEDTools: a flexible suite of utilities for comparing genomic features. Bioinformatics 26:841–842

17. Sedlazeck FJ, Rescheneder P, Smolka M, Fang H, Nattestad M, Von Haeseler A, *et al.* (2018) Accurate detection of complex structural variations using single-molecule

sequencing. Nat Methods 15:461–468. doi: 10.1038/s41592-018-0001-7

18. Serrano J, Snuderl M (2018) Whole Genome DNA Methylation Analysis of Human Glioblastoma Using Illumina BeadArrays. Methods Mol Biol 1741:31–51. doi: 10.1007/978-1-4939-7659-1_2

19. Skidmore Z, Wagner A, Lesurf R, Campbell K, Kunisaki J, Griffith O, *et al.* (2016) GenVisR: Genomic Visualizations in R. Bioinformatics 32:3012–4

20. Uhrig S, Ellermann J, Walther T, Burkhardt P, Fröhlich M, Hutter B, *et al.* (2021) Accurate and efficient detection of gene fusions from RNA sequencing data. Genome Res 31. doi: 10.1101/gr.257246.119

21. Wang K, Singh D, Zeng Z, Coleman SJ, Huang Y, Savich GL, *et al.* (2010) MapSplice: Accurate mapping of RNA-seq reads for splice junction discovery. Nucleic Acids Res 38:e178. doi: 10.1093/nar/gkq622

22. Zhang J, Walsh MF, Wu G, Edmonson MN, Gruber TA, Easton J, *et al.* (2015) Germline Mutations in Predisposition Genes in Pediatric Cancer. N Engl J Med 373:2336–2346. doi: 10.1056/NEJMoa1508054
